# Determinants of Ligand Specificity and Functional Plasticity in Type I Interferon Signaling

**DOI:** 10.3389/fimmu.2021.748423

**Published:** 2021-10-07

**Authors:** Duncan Kirby, Baljyot Parmar, Sepehr Fathi, Sagar Marwah, Chitra R. Nayak, Vera Cherepanov, Sonya MacParland, Jordan J. Feld, Grégoire Altan-Bonnet, Anton Zilman

**Affiliations:** ^1^ Department of Physics, University of Toronto, Toronto, ON, Canada; ^2^ Ajmera Family Transplant Centre, Toronto General Research Institute, Departments of Laboratory Medicine and Pathobiology and Immunology, University of Toronto, Toronto, ON, Canada; ^3^ Department of Physics, Tuskegee University, Tuskegee, AL, United States; ^4^ Sandra Rotman Centre for Global Health, Toronto General Research Institute, University of Toronto, Toronto, ON, Canada; ^5^ Toronto Centre for Liver Disease, University Health Network, Toronto, ON, Canada; ^6^ Immunodynamics Group, Laboratory of Integrative Cancer Immunology, Center for Cancer Research (CCR), National Cancer Institute (NCI), Bethesda, MD, United States; ^7^ Institute for Biomedical Engineering, University of Toronto, Toronto, ON, Canada

**Keywords:** type I interferon (IFN) signaling, functional plasticity, signal processing, cellular decision making, ligand specificity

## Abstract

The Type I Interferon family of cytokines all act through the same cell surface receptor and induce phosphorylation of the same subset of response regulators of the STAT family. Despite their shared receptor, different Type I Interferons have different functions during immune response to infection. In particular, they differ in the potency of their induced anti-viral and anti-proliferative responses in target cells. It remains not fully understood how these functional differences can arise in a ligand-specific manner both at the level of STAT phosphorylation and the downstream function. We use a minimal computational model of Type I Interferon signaling, focusing on Interferon-*α* and Interferon-*β*. We validate the model with quantitative experimental data to identify the key determinants of specificity and functional plasticity in Type I Interferon signaling. We investigate different mechanisms of signal discrimination, and how multiple system components such as binding affinity, receptor expression levels and their variability, receptor internalization, short-term negative feedback by SOCS1 protein, and differential receptor expression play together to ensure ligand specificity on the level of STAT phosphorylation. Based on these results, we propose phenomenological functional mappings from STAT activation to downstream anti-viral and anti-proliferative activity to investigate differential signal processing steps downstream of STAT phosphorylation. We find that the negative feedback by the protein USP18, which enhances differences in signaling between Interferons *via* ligand-dependent refractoriness, can give rise to functional plasticity in Interferon-*α* and Interferon-*β* signaling, and explore other factors that control functional plasticity. Beyond Type I Interferon signaling, our results have a broad applicability to questions of signaling specificity and functional plasticity in signaling systems with multiple ligands acting through a bottleneck of a small number of shared receptors.

## Introduction

Specificity in molecular signaling networks is essential for cells to respond appropriately to changes in their environment. In one important example - an immune response to infection - signal specificity is crucial to ensure the correct cellular responses in immune and other cell types. In a common view of receptor signaling, specificity is encoded in the strength of ligand-receptor binding resulting from the details of molecular ligand-receptor interactions (1). A high affinity ligand typically produces a stronger response while a weak affinity ligand produces little response. In this picture of receptor signaling, each receptor binds its cognate ligand with a much higher affinity than any other ligand to produce an intracellular response uniquely specific to that receptor-ligand pair (2).

However, in many signaling pathways multiple ligands can act through overlapping sets of receptors or downstream intracellular signaling molecules, and yet induce distinct cellular responses. Overlapping components between signal pathways is known as crosstalk, and this feature has been observed in cytokine signaling through the Jak/STAT pathway, the TGF superfamily of ligands acting through the SMAD pathway, a variety of ligands acting through the NF-*κ*B pathway, and others (3–8). These examples challenge the idea of specificity based purely on ligand-receptor binding pairs because the same level of receptor occupancy can be achieved by either a low concentration of a high affinity ligand or by a high concentration of a low affinity ligand (9). The ability of a signaling pathway to generate distinct cellular responses in the presence of crosstalk has been termed functional plasticity (10, 11). Crosstalk without distinct cellular responses generates redundancy between ligands, which may be useful for other reasons (12).

In this paper we focus on the following question: how can functional plasticity arise in systems where ligands act through *the same receptor*? This is an extreme form of crosstalk since ligands necessarily activate the same set of downstream intracellular molecules.

**Figure 1:**
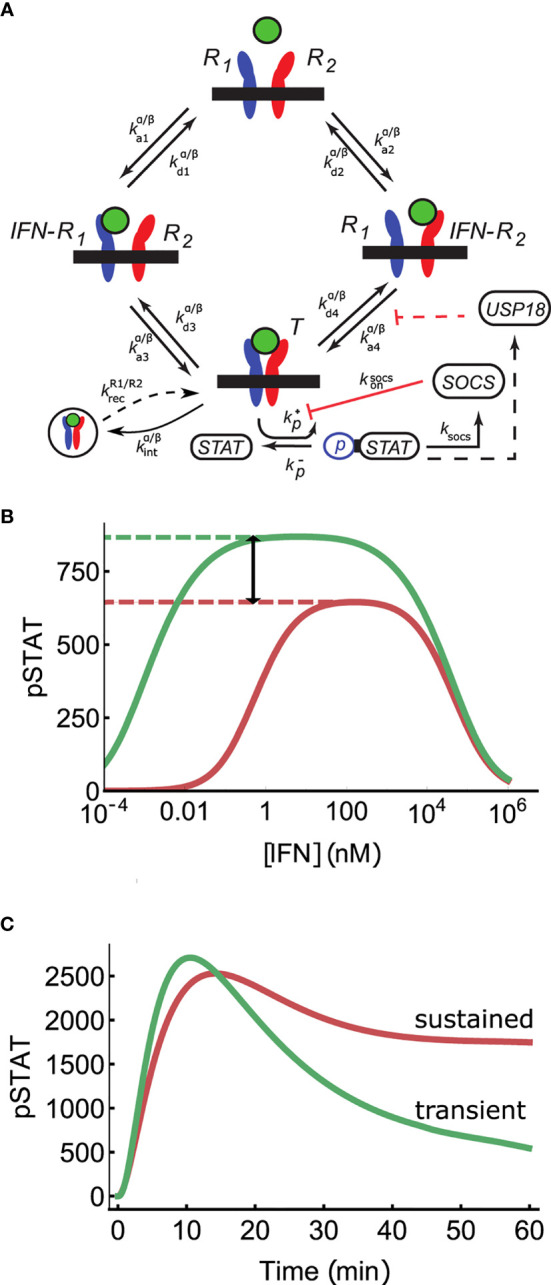
Mechanisms of signal discrimination. **(A)** Interferon induced receptor dimerization can occur *via* two paths. In the first, an IFN molecule (green circle) binds IFNAR1 (R_1_ in the diagram), and IFNAR2 subsequently associates with the binary IFN-R1 complex, leading to the formation of the ternary complex T. In the second pathway, the sequence of the association events is reversed. The multi-step process of STAT phosphorylation is combined into an aggregate rate *k_p_*, as are all steps leading to SOCS1 production in rate *k_socs_*. SOCS1 binds to IFNAR1 independent of whether or not IFNAR1 is bound by IFN, and inhibits phosphorylation of STAT, with rate *k_socs on_*. USP18 is modeled as pre-bound to IFNAR2 (i.e., before IFN stimulation) and reduces IFNAR1 recruitment into the ternary complex by increasing k_-4_ (see *Long Time Deactivation via USP18 Regulates Receptor Complex Stability to Achieve Absolute Discrimination*). **(B)** Absolute discrimination encodes signal identity through differences in the maximum response amplitude for weaker binding IFN*α*2 (red) compared to stronger binding IFN*β* (green) even for saturating doses ([IFN] >*K_eff_*). Thus, if the pSTAT response is greater than this threshold (red dashed line) the signal is unambiguously identified as belonging to IFN*β*. The region of absolute discrimination is indicated by black arrow. **(C)** Time course discrimination differentiates ligands based on, for example, sustained (red, IFN*α*2) *versus* transient (green, IFN*β*) pSTAT levels.

Examples of such crosstalk include biased agonism in G-protein coupled receptors, and chemokine and cytokine signaling in the immune system (8, 10, 13–15). We use the prototypical example of the Type I Interferon (IFN) family of cytokines to study ligand crosstalk through a shared receptor (**Figure 1A**) (16–18). Less extreme forms of cross-talk may be found, for example, in the partial overlap of types of STATs activated by other cytokines in Jak/STAT signaling (19, 20). In this work, we focus on the Type I IFN signaling because – beyond its fundamental biological importance – the bottleneck of completely overlapping receptors and proximal signaling factors activated by functionally distinct cytokines makes it a good system to study signal specificity.

Type I IFN signaling is an essential component of the early innate immune response to viral infections, and recently the IFN anti-viral response has received a great deal of attention due to its role during COVID-19 infection and other viral infections (21–26). IFN signaling is also important in other diseases including a number of cancers and multiple sclerosis (16, 27). Different IFNs are known to play different roles in the immune response to infection as well as in other diseases, and different IFN subtypes are targeted in clinical applications (8, 10, 28, 29). These many roles, and their varying significance among IFN subtypes, make Type I IFN signaling an important model to study crosstalk between functionally distinct ligands acting through the same receptor.

As a brief overview, the Type I IFN family of signaling molecules is composed of thirteen subtypes of IFN-α, IFN-β, IFN-ϵ, IFN-κ and IFN-ω (29). All these cytokines act through the same Type I IFN receptor and induce expression of Interferon Stimulated Genes (ISGs). These ISGs drive diverse functions, with anti-viral and anti-proliferative cellular assays traditionally being used to classify functional differences between ligands, although the phenotypes resulting from ISG induction are diverse and the division of ISGs into these classes is not strictly binary (30–32).

The Type I IFN receptor is composed of two subunits, IFNAR1 and IFNAR2 (17) (see **Figure 1A**). The cytoplasmic domain of IFNAR2 is constitutively bound by the tyrosine kinase Jak1, while IFNAR1 is pre-associated with Tyk2 (29). When an IFN molecule binds to the extracellular domain of one or the other of these receptor subunits, the bound complex then recruits the remaining receptor subunit *via* binding to the IFN molecule. Once this ternary complex is formed, the proteins known as Signal Transducer and Activator of Transcription (STAT) 1 and STAT2 are recruited to the receptor complex. These STATs are phosphorylated and dimerize to form a pSTAT1-pSTAT2 heterodimer (See **Figure 1A**). The exact order and molecular details of STAT recruitment, phosphorylation, and dimerization are not fully known (11). This dimer binds IRF9, another transcription factor, and the resulting complex is imported to the nucleus to activate transcription of a variety of IFN regulated genes. Phosphorylation of additional STATs (e.g. STAT3 and STAT5) and alternate STAT dimers (e.g. STAT1 homodimers) have also been reported, although functional roles distinct from pSTAT1-pSTAT2 dimers have yet to be assigned to these complexes (29, 33).

IFN*β* and IFN*α*2 are commonly chosen IFN subtypes to study signaling crosstalk because they are well separated in terms of their anti-proliferative and anti-viral activities (11, 18, 34, 35). Specifically, while anti-viral potencies are similar between all Type I IFNs, IFNβ elicits a much stronger anti-proliferative response compared to IFN*α*2 both in terms of the concentration required for half-maximal inhibition (its IC_50_, which is a few picomolar for IFN*β* compared to nanomolar for IFN*α*2) and in terms of the maximal anti-proliferative response (although this difference varies across cell types) (10, 11, 34–39).

These differences in anti-proliferative and anti-viral potencies might naturally be assumed to arise from differences in the induced pSTAT response. Unfortunately, in many cases only the relative pSTAT responses (39, 40), or only the pSTAT EC_50_ (38, 41), or only semi-quantitative measures of the pSTAT response (10, 34–36, 42) are reported. It is therefore difficult to determine with certainty if differences in the pSTAT response consistently explain differences in anti-viral and anti-proliferative potencies between IFNs. However, most factors which have been suggested to play a role in the emergence of functional plasticity in Type I IFN signaling regulate the pSTAT response.

The primary factor differentiating IFNs is their affinity for the receptor subunits (18, 39, 40, 43–45). Previous studies have reported that, in human cells, IFN*β* binds the IFNAR2 subunit much stronger than IFN*α*2 (10). Furthermore, mutation studies have demonstrated that much of the functional difference between these two IFNs can be recovered by IFN*α*2 mutants which bind the IFNAR2 subunit with a similar strength to wild-type IFN*β* (38). However, ligand affinity cannot be the only important factor for functional plasticity since the anti-viral and anti-proliferative potencies of different IFN subtypes scale differently with ligand affinity (29).

Other factors have also been shown to be influential in tuning the functional differences between IFNs. These include differential expression of the receptor subunits on the cell surface (35, 37), differential receptor internalization induced by IFN stimulation (36, 38, 39, 46, 47), negative feedback on the signaling system by upregulation of the protein Suppressor of Cytokine Signaling (SOCS) 1 in response to IFN stimulation (48, 49), and response refractoriness *via* upregulated expression of the inhibitory protein USP18 (10, 46, 48, 50–52). How all these factors act in concert to regulate specificity is still not clear (11, 29).

Signaling systems with crosstalk must decouple ligand concentration from ligand affinity in the downstream response if functional plasticity is to be achieved. We refer to such a decoupling as *absolute discrimination* (53, 54). In systems with absolute discrimination, functional differences can only be partially compensated by changes in ligand concentration. We briefly outline possible biological mechanisms to achieve absolute discrimination.

Amplitude-based absolute discrimination encodes ligand affinity into the magnitude of receptor outputs *via* affinity-dependent saturation of the receptor (see **Figure 1B**). This means that there is a threshold level of receptor activation which only sufficiently high affinity ligands can surpass, even at high ligand concentrations. Cellular responses that require receptor activation above this threshold are consequently specific to high affinity ligands. One proposed mechanism for affinity-dependent receptor saturation relies on the non-monotonic nature of the dose-response curve of dimeric receptors (53, 55). Kinetic proofreading and its enhanced version know as adaptive sorting, studied in the context of T cell receptor sensing, also demonstrate affinity-dependent saturation (54, 56). These mechanisms are therefore capable of amplitude-based absolute discrimination of signals.

Another mechanism for decoupling ligand concentration from ligand affinity is based on time course discrimination, which separates signals based on differences in the temporal pattern of receptor activity. One commonly considered mechanism is to differentiate transient from sustained receptor activation (**Figure 1C**). Time course discrimination has been proposed in ERK and NF-*κ*B signaling as well as in responses to metabolic and environmental stressors (57–60).

A third mechanism for absolute discrimination is combinatorial encoding, where different ligands induce different compositions of receptor outputs (60, 61). This mechanism appears to be most useful for decomposition of mixtures of ligands, a signal processing problem which is not treated here. For this reason, combinatorial encoding remains outside the scope of this investigation and will be addressed in future work.

In this paper we address how functional plasticity can arise in Type I IFN signaling, where many cytokines act through the same receptor. We present a model of Type I IFN signaling which incorporates the essential factors previously suggested to be important for signal specificity. Using measurements of the pSTAT response in primary mouse B cells stimulated with IFN*α*2 or IFN*β* as well as previously published results, we validate our model at the level of the receptor activation and the downstream STAT response. We quantify differences in the pSTAT response between IFN*α*2 and IFN*β* and clarify the differing roles of various feedback factors. We find that negative feedback by the protein USP18, which enhances differences in signaling between IFN*α*2 and IFN*β*, can achieve amplitude-based absolute discrimination. Finally, we develop phenomenological models to demonstrate how functional plasticity in anti-viral and anti-proliferative activity can arise from this mechanism.

## Methods

### Experimental Methods

Single cell suspensions of mouse splenocytes were prepared as follows. Spleens were harvested from 4-month-old female C57BL/6J mice (Strain 00064 from Jackson Laboratory, Bangor ME) and mechanically dissociated with a sterile syringe plunger. We performed a 1 min ACK lysis to remove any red blood cells. Splenocytes were then washed in PBS and exposed to a solution of 0.1M glycine in PBS (with adjusted pH at 4.0) for 1min on ice, followed by 2 washes with complete medium. Cells were then rested in complete medium for 2 hours at 37°C.

Cells were then exposed to a serial dilution of IFN-*α*2 or IFN-*β* (R&D systems, Minneapolis MN) in complete medium and incubated for varied amounts of times. At each time point, ice-cold 4% paraformaldehyde solution was added to the reaction culture (for a final concentration of 1.6% paraformaldehyde) and cells were left in fixative for 15min on ice. Cells were then spun, their supernatants were discarded, and cells were then permeabilized with a 15-min incubation in 90% methanol on ice. Cells were then washed twice with FACS buffer (4% FBS in PBS + 0.1% sodium azide) and stained with primary anti-phospho-STAT1 (clone 58D6 from Cell Signaling Technologies, Danveres MA) for 30min at room temperature. Cells were then washed once with FACS and stained with a cocktail of antibodies against surface markers and Fab anti-Rabbit secondary antibody (see **Table S1**) for 30min at room temperature. Cells were then washed once with FACS buffer and resuspended in FACS buffer with DAPI (1µMol) and acquired on a 20-channel Fortessa cytometer (BD Biosciences). Single cell gating for live CD19+ B cells and levels of phosphorylated STAT1 were exported as geometric mean for further analysis (**Figures S1, S2**).

### Mathematical Methods

**Figure 1A** illustrates a streamlined model of IFN which includes receptor assembly, STAT phosphorylation, receptor internalization, and SOCS1 inhibition. The model aggregates various biochemical sub-steps, such as STAT association and dissociation with active receptor complexes, into single-step reactions. Since only pSTAT1 was measured experimentally, our model does not describe STAT1-STAT2 dimers explicitly but rather uses a single STAT variable which can be phosphorylated to an active pSTAT form. Since the JAK kinases are constitutively bound to their corresponding receptor subunits, we do not model them explicitly and instead consider the ternary complex to phosphorylate STAT to pSTAT (28). A more detailed model which explicitly models additional biochemical steps is presented in the SI and yields similar predictions for the pSTAT response (**Figure S3**), indicating that the minimal model of **Figure 1** encapsulates the salient features of the signaling network. We include basal and IFN-induced receptor internalization in our model as well as recycling of the receptor subunits back to the cell surface, assuming that the internalized ligand is degraded (36). The model includes negative feedback by SOCS1, which is produced downstream of pSTAT and binds IFNAR1 to block STAT phosphorylation. It is known that IFNAR1 and IFNAR2 surface expression levels vary between cells, and in practice we found it necessary to model this variation using a log-normal distribution of expression levels for IFNAR1 and IFNAR2 (62). All model predictions are made by taking 30 samples from these receptor distributions and averaging the predicted pSTAT responses. This is a simple way to model heterogeneity of receptor expression, a factor we explore more in depth below.

The complete mathematical description of our model using a system of ordinary differential equations (ODEs) is provided in **Tables S2, S3** and is solved numerically using the PySB python package (63). We used ligand-receptor binding and unbinding rates measured for human IFNs and applied detailed balance to obtain in-membrane rates (see **Table S2**) (10, 38). A global scale factor of 1.5 was applied to convert the number of pSTAT molecules predicted by the model into units of MFI measured by experiment. Estimates of the values of model parameters which have not been directly measured were obtained using the systems biology package PyDREAM to perform Markov Chain Monte Carlo (MCMC) simulations (**Figure S4**) (64). The mean absolute error (MAE) between the data and the average prediction of our best fit model is 40%. A bootstrap analysis of the fitting procedure using an 80-20 train-test split of our experimental data yielded a similar MAE across bootstrap batches, further validating our parameter fit and demonstrating the robustness of the fitting procedure.

To fit the phenomenological models of anti-viral and anti-proliferative activity to the data in Section *Proximal Receptor Signaling Maps to Biological Activity*, we used standard nonlinear function fitting procedures provided by the *scipy* Python package which are based on the Trust Region Reflective algorithm. The fitting procedure for Eq. 5-6 yielded a root mean square error of 11%.

Many aspects of IFN signaling are captured by the relatively simple equilibrium description of receptor complex formation obtained from the steady state of our ODE model, providing qualitative insight into how various components of this system affect signaling dynamics. The equilibrium solution for the number of active ternary complexes formed in response to a ligand can be found by solving the chemical kinetic equations along with the detailed balance condition *K*_1_ *K*_3_ = *K*_2_ *K*_4_ (see below), where *K*_1_ and *K*_2_ are the equilibrium dissociation constants for IFN binding to each of IFNAR1 and IFNAR2, and *K*_4_ and *K*_3_ are the in-membrane dissociation constants for recruitment of IFNAR1 and IFNAR2 respectively to the ternary complex (see **Figure 1A**) (53). The result is an expression for the equilibrium number of ternary complexes (53, 65):


(1) $$T=\frac{R_{T}}{2}(\Xi (I)-\sqrt{\Xi {\left(I\right)}^{2}-1+{\left(\Delta /R_{T}\right)}^{2})}$$

where $\Xi (I)=1+\frac{K_{4}}{R_{T}}\frac{\left(I+K_{2})(I+K_{1}\right)}{I\;K_{1}}$ , *I* is the extracellular IFN concentration, $\text{Δ}=R_{1}^{T}-R_{2}^{T}$ is the difference between the (two-dimensional) cell surface densities of *R*_1_ (IFNAR1) and *R*_2_ (IFNAR2), $R_{T}=R_{1}^{T}+R_{2}^{T}$ is the total surface density of receptors of both types. The detailed balance condition arises from the fundamental physics relating the dissociation constants to the binding energies of the ligand with the receptor subunits, *E*_1_ and *E*_2_. In the simplest case, $K_{1}=\frac{1}{v}e^{-\frac{E_{1}}{kT}}$ and $K_{2}=\frac{1}{v}e^{-\frac{E_{2}}{kT}}$ , where *v* is a small volume roughly proportional to the average volume of the reacting molecules; for three-dimensional binding affinities, it is customary to set *v* = 1*M* ^–1^ ≈ 1.75*nm* ^3^ (1, 66). The in-membrane affinities *K*_3_ and *K*_4_ obey the same physics; in the ideal case where the ligand binding to one of the subunits does not affect its binding to the other one, $K_{4}=\frac{1}{a}e^{-\frac{E_{1}}{kT}}$ and $K_{3}=\frac{1}{a}e^{-\frac{E_{2}}{kT}}$ where *a* is the average area of the reacting molecules (67). The detailed balance condition also holds for more complicated dependencies of the dissociation constants on the molecular interaction energies as long as the binding-unbinding reactions involved in the receptor dimerization do not require direct input of metabolic energy (68).

**Figure 2:**
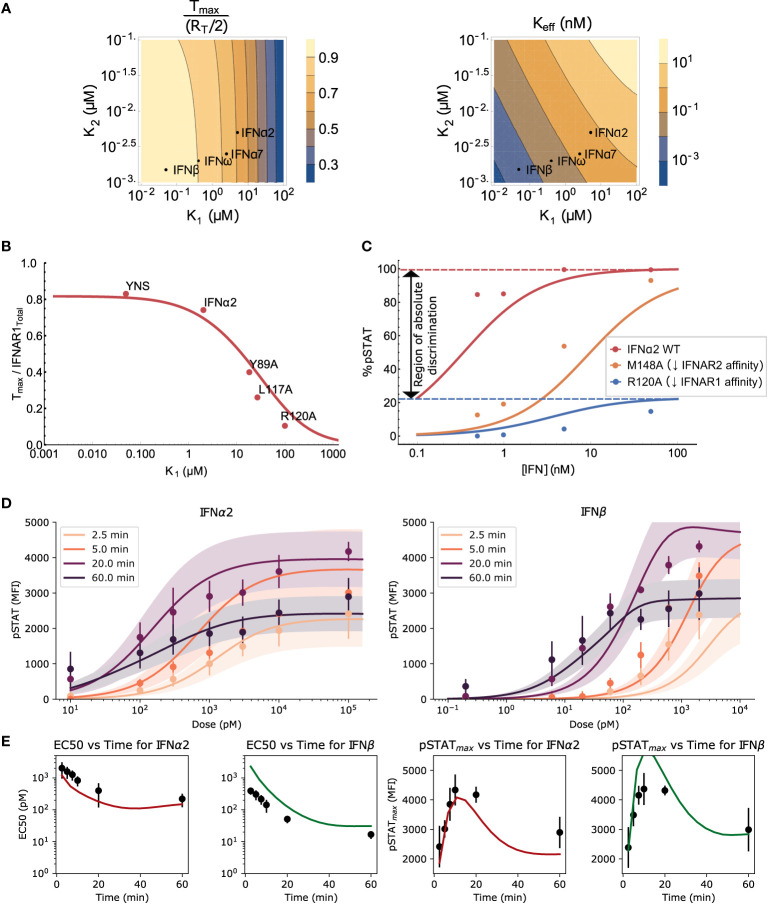
Validation of the model and the effects of ligand affinity on response specificity. **(A)** The theoretically predicted values of *T_max_* and *K_eff_* as functions of binding affinities, maintaining detailed balance (see *Mathematical Methods*). *T_max_* is normalized to be between 0 and 1, with Δ = 0 in both plots. Both *T_max_* and *K_eff_* are sensitive to changes in *K*_1_ but only *K_eff_* is sensitive to changes in *K*_2_ in the regime of interest. **(B)** The *T_max_* normalized by total IFNAR1 for different IFN*α*2 mutants was measured directly in (10). Varying IFNAR1 binding affinity in Eq. 1 (red curve) explains observed differences in *T_max_* (points). **(C)** pSTAT1 Western blot band intensities (points) measured in (10) for wild-type (WT) IFN*α*2 and the IFN*α*2 mutants R120A and M148A. The effect of the mutations on the dose-response curve are recapitulated by the equilibrium model (Eq. 4) using a 60-fold (R120A) and 50-fold (M148A) change in *K*_1_ (and the corresponding change in *K*_4_) or *K*_2_ (and *K*_3_), respectively. Kinetic parameters (*K_p_*, *S_T_*, *A*) match those used in the ODE model presented in **(D)**, and affinities from (10). **(D)** The experimental data from mouse B cells (points with error bars) and simulated (solid lines) dose response curves for IFN*α*2 and IFN*β* at different stimulation durations. Each dose-response curve is the average model prediction with a one standard-deviation envelope, representing 30 samples from the fitted log-normal distribution of IFNAR1 and IFNAR2 expression. Additional timepoints shown in **Figure S2**. **(E)** The EC_50_ (left) and maximal response (right) computed from the data (black points with std. dev. shown) and the model (solid line).

The ternary complex dose-response curve is a non-monotonic function of the ligand concentration. The maximal response *T_max_* is attained at $I_{m}=\sqrt{K_{1}K_{2}}$ . Crucially, weak affinity ligands saturate to a lower maximum receptor activity than high affinity ligands. At concentrations below the maximal response, the dose-response curve can be approximated with a Michaelis-Menten type function:


(2) $$T\approx T_{\max}\frac{I}{K_{eff}+I}$$

where *K_eff_* is the effective equilibrium dissociation constant of ternary complex formation, with *K_eff_* given by:


(3) $$K_{eff}-\frac{K_{1}}{8}(D-\sqrt{D^{2}-64X})$$

where $D=\frac{R_{T}}{K_{4}}+1+10X^{\frac{1}{2}}+X+\frac{3R_{T}}{K_{4}}\sqrt{B^{2}-1+{\left(\Delta /R_{T}\right)}^{2}},B=1+(K_{4}/R_{T}){\left(1+\sqrt{X}\right)}^{2}$ and *X* = *K*_2_/*K*_1_. It is important to note that Equations (1-3) predict contours of constant *K_eff_* and *T_max_* as a function of receptor binding strengths, as shown in **Figure 2A**.

The product of the dissociation constants *K*_1_ × *K*_2_ has been empirically observed to correlate well with *K_eff_* for a wide range of IFNs (39). This product is therefore expected to be approximately proportional to related biological *IC*_50_ values and other quantities related to *K_eff_*, although there is a systematic bias in using *K*_1_ × *K*_2_ in place of *K_eff_* (see also **Figure S5**).

Ternary complex formation leads to the production of phosphorylated STAT1-STAT2 dimers at rate $k_{p}^{+}$ , which are constitutively de-phosphorylated at rate $k_{p}^{-}$ . In the limit that intracellular STAT equilibrates with the active receptor complexes faster than STAT is phosphorylated, the equilibrium copy number of pSTAT1-pSTAT2 dimers is well approximated by a Michaelis-Menten equation:


(4) $$pSTAT=S_{T}\frac{TA}{K_{p}+TA}≅S_{T}\frac{T_{\max}}{T_{\max}+K_{p}/A}\frac{I}{I+K_{eff}\text{/(}1+T_{\max}A/K_{p})}$$

where *S_T_* is the total number of STAT molecules in the cell, $K_{p}=k_{p}^{-}/k_{p}^{+}$ , and *A* is the cell surface area. Thus, the maximal magnitude of the pSTAT response is $pSTAT_{\max}=S_{T}\frac{T_{\max}}{T_{\max}+K_{p}/A}$ and the half-maximal response of pSTAT is *pSTAT EC*_50_ = *K_eff_* /(1 + *T_max_A*/*K_p_*). Unlike the effective affinity of the receptor binding *K_eff_*, the *EC*_50_ of the pSTAT response explicitly depends on the cell area and not only on the surface densities of the receptor sub-units.

## Results

### Validation of the Model and the Effects of Ligand Affinity on Response Specificity

To validate our model at the level of receptor assembly (Equation 1), we compared the predicted number of ternary complexes for a variety of IFN*α*2 mutants to previously reported measurements that used single-molecule tracking of receptor subunits, as reported in (10). IFNAR1 affinity has been shown to be the main differentiating factor between these IFNs (39, 43, 69). We demonstrate in **Figure 2B** that differences in the level of maximum ternary complex formation between IFNs are well explained in our model of receptor assembly using only the differences in IFNAR1 binding strength between these IFNs [i.e., holding IFNAR2 binding strength constant at the measured IFN*α*2 wild type level (38)]. This agreement between the model and data for a wide variety of affinity-altering mutations of the IFN molecule demonstrates that our model captures the essential features of receptor assembly kinetics in Type I IFN signaling.

Further model validation is provided by looking at the effect of two mutations to IFN*α*2 on the pSTAT dose-response curve. The mutants IFN*α*2-R120A and IFN*α*2-M148A have reduced affinities for IFNAR1 and IFNAR2 respectively and have been used to explore the dependence of the pSTAT response on IFN-receptor binding strength (10). Mutations of the IFN ligand alter the binding energy and therefore the dissociation rates of all reactions involving IFN-receptor interaction due to the detailed balance condition described above. In particular, reduction in IFNAR1 affinity alters both *K*_1_ and *K*_4_, and a reduction in IFNAR2 affinity alters *K*_2_ and *K*_3_ (see *Mathematical Methods*). The IFN*α*2-R120A ligand, with a 60-fold reduced affinity for IFNAR1, was shown to alter both *pSTAT_max_* and the pSTAT *EC*_50_ while the IFN*α*2-M148A ligand, with a 50-fold reduced affinity for IFNAR2, only affected the pSTAT *EC*_50_. In **Figure 2C** we show that our equilibrium model recapitulates the effect of these changes in binding energy on the pSTAT dose-response curves. The difference in the effects of these two mutations is not surprising considering we already showed in **Figure 2A** that, in the range of *K*_1_ and *K*_2_ affinities observed for Type I IFNs, *T_max_* – and therefore *pSTAT_max_* – is largely insensitive to changes in IFNAR2 affinity while *K_eff_* – and therefore *pSTAT EC*_50_ – is sensitive to both IFNAR1 and IFNAR2 affinity. The difference in *pSTAT_max_* between IFN*α*2 and IFN*α*2-R120A, shown in **Figure 2C**, demonstrates that ligand affinity is sufficient to enable amplitude-based absolute discrimination. This effect has also been observed in other signaling pathways (55, 70, 71). Additionally, the results in **Figures 2A–C** suggest that *pSTAT_max_* and the pSTAT *EC*_50_ can be tuned independently by ligand binding strengths. We will return to this point in the Discussion.

We next focused on the pSTAT response to IFN*α*2 and IFN*β* in particular, explicitly including the effects of short-term and long-term negative feedbacks in order to capture changes in the response over time. To this end, we fit the free parameters of our ODE model (the rates of STAT phosphorylation/dephosphorylation, of SOCS1 production, of receptor internalization, and receptor subunit expression levels, summarized in **Table S3**) to our measurements of primary mouse B cell response to IFN stimulation (see *Methods* for details). **Figure 2D** shows that our model can be fit closely to our experimental data. The MCMC fitting did not tightly constrain the free model parameters, which is to be expected due to “sloppiness” observed in most systems biology models as a result of multiple compensatory feedbacks in these systems (72, 73).

Both the *EC*_50_ and *pSTAT_max_* of the pSTAT dose-response curve depend on the stimulation time but converge at long times to steady state values for each IFN, and the values predicted by the ODE model are in agreement with the experimentally determined ones (**Figure 2E**). Interestingly, we do not observe a significant difference in the *pSTAT_max_* between IFN*α*2 and IFN*β*. This is somewhat surprising because there is a noticeable difference in the expected *T_max_* between these IFNs (as shown in **Figures 2A, B**), and furthermore our equilibrium model naïvely predicts an approximately 15% lower *pSTAT_max_* according to Eq. 4, for reasonable choices of parameters (*R_T_* = 2000 subunits, *A* = 450 *μm* ^2^, affinities and *K_p_* from **Table S3**). The fold-change in *pSTAT_max_* between IFNs can be estimated from Equation 4 as


$$\frac{pSTAT_{\max}^{\alpha }}{pSTAT_{\max}^{\beta }}=\frac{T_{\max}^{\alpha }}{T_{\max}^{\beta }}\left(\frac{1+T_{\max}^{\beta }/(K_{p}/A)}{1+T_{\max}^{\alpha }/(K_{p}/A)}\right)$$

For large *K_p_*/*A*, the difference in response is expected to be the same as the difference in *T_max_*. In contrast, for small *K_p_*/*A* no difference in *pSTAT_max_* is expected despite a difference in *T_max_*. As we will show below, additional factors besides the cell surface area and phosphorylation constant (*A* and *K_p_*) also affect the difference in *pSTAT_max_*. We note here that the lack of a region of absolute discrimination between IFN*α*2-induced and IFN*β*-induced pSTAT response in our experimental results in primary mouse B cells is consistent with some of the measurements performed in a variety of human cell types exposed to IFN*α*2 and IFN*α*2-YNS, an IFN*β* mimic (39). On the other hand, there are also reports of differences in maximal pSTAT response between IFN subtypes in other settings (35, 39, 42). This is reasonable since different cell types have different values for *K_p_*, *A*, and other feedback rates which may tune the difference in *pSTAT_max_*. Thus, in the sections below we explore additional factors besides ligand affinity which can regulate the pSTAT response and allow for functional plasticity.

### Receptor Expression Levels Can Regulate Signaling Specificity

**Figure 3:**
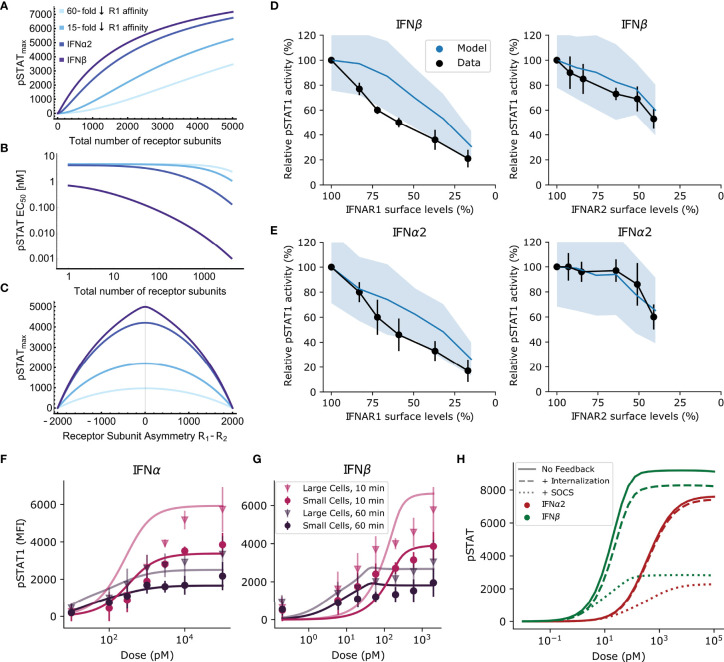
Receptor expression levels can regulate signal specificity. **(A, B)** The equilibrium model predicts how *pSTAT_max_* **(A)** and *pSTAT EC*_50_ **(B)** vary with the total number of receptor subunits on the cell surface. From violet to lightest blue, lines correspond to high affinity IFN*β*, lower affinity IFN*α*2, and IFN*α*2 mutants with 15-fold and 60-fold lower IFNAR1 affinity, respectively (see text). **(C)** The dependence of the response on the difference in expression of R1 and R2 subunits for each of the IFN subtypes from A&B. The results in **(A–C)** are similar for the computational model (not shown); see text. **(D, E)** pSTAT1 levels following siRNA transfection to knockdown IFNAR1 or IFNAR2 and subsequent stimulation for 45 minutes with 200 pM of IFN. Black: Western blot band intensity measurements; Blue: predicted response from model. Responses are normalized by maximum response. Model uses measured human IFN affinities and mean receptor density between 0.1 and 1 molec. *μ*m^-2^, consistent with measurements for WISH cells (10). **(F, G)** Points: Experimental flow cytometry data from mouse B cells with one std. dev. Lines: simulated average pSTAT responses for IFN*α*2 **(F)** and IFN*β* **(G)** using the same model parameters as . More copies of IFNAR are expressed in large cells, but no significant difference between the maximum pSTAT response to IFN*α*2 and IFN*β* is observed. **(H)** Solid lines: simulated pSTAT dose-response curve at 60 minutes without any negative feedback (i.e., setting SOCS1 binding and receptor internalization rates to zero). Dotted lines: the effect of SOCS1. Dashed lines: the effect of receptor internalization. In both cases the dose-response curve is diminished by these negative feedbacks. The difference in *pSTAT_max_* between IFN*α*2 (red) and IFN*β* (green) remains constant with the action of SOCS1 inhibition while the difference between IFN*α*2 and IFN*β* is diminished by receptor internalization.

Receptor density on the cell surface has been suggested as an important factor affecting the specificity of response (35, 74, 75). As shown in **Figure 3A**, the theoretically predicted separation between *pSTAT_max_* for ligands of different affinities (i.e., the region of absolute discrimination) remains nearly constant except at very low receptor expression levels – because the dominant effect of a change in the number of the surface receptors is the overall increase in the number of ternary complexes. For this reason, total receptor expression is not expected to affect amplitude-based absolute discrimination.

By contrast, as shown in **Figure 3B**, the theoretically predicted *pSTAT EC*_50_ decreases monotonically with increasing total number of receptor subunits and can shift by several orders of magnitude over a biologically realistic range of receptor expression levels [e.g. 10^2^ *versus* 10^3^ copies, (18)]. This suggests that modulation of total IFNAR1 and IFNAR2 expression levels could make a cell preclude weaker binding IFNs from activating downstream responses that require high intracellular pSTAT levels. This is conceptually similar to absolute discrimination but without complete decoupling of ligand concentration from the pSTAT response. For physiological numbers of receptor subunits (e.g., ~10^3^ total receptor subunits), preventing a high pSTAT-dependent response to IFN*α*2 would require IFN concentrations to remain well below 1 nM at all times. Note, however, that this does not provide discrimination between two distinct ligands requiring different cellular responses because the weaker binding ligand is effectively never detected. Rather, this effect could provide a way of selectively responding *only* to high affinity ligands as a function of receptor expression levels. This could be used to selectively target specific cell types, a point we return to in the *Discussion*.

**Figure 3C** shows that asymmetric expression of IFNAR1 and IFNAR2 is predicted to be an important factor controlling the difference in response for different ligands. The greatest degree of separation in *pSTAT_max_* is achieved for equal levels of IFNAR1 and IFNAR2, and this separation decreases monotonically and symmetrically for a growing imbalance in expression levels. The symmetry in the effect of expression imbalance may seem counter-intuitive given that IFNAR1 affinity is the major discriminant between IFN subtypes. However, Eq. 1 shows that the response only depends on IFNAR1 abundance relative to IFNAR2 abundance (i.e., through the terms *R_T_* and Δ), so the separation in *pSTAT_max_* between different IFNs remains nearly constant unless an imbalance between IFNAR1 and IFNAR2 is introduced.

Although we could not directly probe the effect of tuning receptor subunit expression on the pSTAT response in primary mouse B cells, this effect has been measured in WISH cells by siRNA knockdown experiments (45). In **Figures 3D, E** we compare the measured and simulated pSTAT1 response after siRNA transfection and subsequent treatment with either IFN*β* analog IFN*α*2-YNS or IFN*α*2. Both model and data exhibit a reduction in pSTAT level that varies approximately linearly with the reduction in IFNAR1 levels for both IFNs. In comparison, the reduction in pSTAT is lower at equivalent levels of IFNAR2 knockdown in both model and experiment. The good agreement between our model and data, especially considering the difference in cell type between the siRNA experiments and those used to parameterize our model, demonstrates that our computational model captures essential factors regulating IFN signaling.

To further investigate the dependence of *pSTAT_max_* and *pSTAT EC*_50_ on the receptor subunit numbers, we used the cell size as a proxy for the receptor number. We grouped B cells from our flow cytometry experiments by their forward scatter size as a proxy for cell size. Cells in the top 20^th^ percentile in forward scatter (i.e., large cells) were experimentally observed to have a greater pSTAT MFI in response to both IFNs compared with those in the bottom 80^th^ percentile (**Figures 3F, G**). To compare experimental data with our model quantitatively, we varied the cell size in our model while keeping the surface concentration of receptor subunits and the cytoplasmic concentration of STAT molecules constant (76). The experimentally observed separation in the *pSTAT_max_* in small and large cells was explained in our model by assuming fixed effective cell radii of 6.5 *μ*m and 8 *μ*m (corresponding to roughly two-fold difference in the cell volume) for the small and large cells respectively. These cell groups may correspond to cells in different phases of the cell cycle or other factors which affect cell size (77). Importantly, none of the rate constants for the biochemical reactions in our model needed to be adjusted from our original fitting procedure (in **Figure 2D**).

### The Role of Negative Feedbacks in Signal Specificity

Both receptor internalization and feedback by SOCS1 have been discussed in the literature as factors relevant for functional plasticity (36, 38, 39, 46–49). Our computational model allows us to compare the role of these two short-term feedback mechanisms in Type I IFN signaling. Since both receptor internalization and SOCS1 upregulation happen rapidly in response to IFN stimulation, there may be partial redundancy in their roles as negative feedback mechanisms (36, 78).

It has been shown that the rate of receptor internalization may be significantly increased in response to IFN stimulation and that this effect is much stronger in response to IFN*β* than to IFN*α*2 (36, 39). These works additionally showed that the rates at which IFNAR1 and IFNAR2 are recycled back to the cell surface may also differ. As a result, each IFN induces a ligand-specific change in the levels of IFNAR1 and IFNAR2 present on the cell surface post-stimulation. This suggests that receptor internalization could be a mechanism for regulating functional plasticity, since receptor expression asymmetry is expected to affect signal separation by amplitude-based absolute discrimination (Section *Receptor Expression Levels Can Regulate Signaling Specificity* and **Figure 3C**). In contrast to receptor internalization, SOCS1 inhibits Tyk2 phosphorylation of STAT1, reducing the number of ternary complexes which are actively phosphorylating STAT. We expect this to have a similar effect to reducing total receptor expression, which was predicted not to regulate signal specificity (Section *Receptor Expression Levels Can Regulate Signaling Specificity*, **Figures 3A, B**).

To test if receptor internalization and SOCS1 play non-redundant roles in regulating response specificity, we used our computational model to query how these feedbacks each regulate the course of the pSTAT response. In **Figure 3H** we plot the predicted dose response curve for IFN signaling without SOCS1 or receptor internalization. By then looking at the isolated effect of SOCS1, we find that both the IFN*α*2 and IFN*β* dose-response curves are reduced by similar amounts by the action of SOCS1. The difference in *pSTAT_max_* between IFNs remains proportionally the same because SOCS1 inhibits STAT phosphorylation rather than ternary complex formation, so that feedback on the system is not dependent on ligand identity. This suggests that SOCS1 does not play a role in the emergence of functional plasticity *via* amplitude-based signal discrimination.

In contrast to the effect of SOCS1, the effect of receptor internalization on the difference in *pSTAT_max_* between IFNs could be significant for amplitude-based signal discrimination. This can be seen in **Figure 3H** by comparing the response predicted by our computational model without any feedback to that with internalization. Receptor internalization rates can play a role in the emergence of functional plasticity because they are specific to the type of bound IFN. It has been reported that receptor internalization is greater for IFN*β* than IFN*α*2, and the parameter fitting procedure for our ODE model also yielded greater receptor downregulation in response to IFN*β* than IFN*α*2 (36). Since in the absence of feedback the *pSTAT_max_* of the dose-response curve at 60 minutes is predicted by our ODE model to be greater for IFN*β* than IFN*α*2 (see **Figure 3H**), and since receptor internalization decreases each pSTAT dose-response curve, greater receptor internalization following IFN*β* stimulation effectively acts to *reduce* the difference in *pSTAT_max_* between these IFNs. In particular, faster IFNAR2 recycling in response to IFN*α*2 relative to IFN*β* [as has been reported in (36)] diminishes the difference in *pSTAT_max_* which might occur due to differences in receptor affinity. This means that receptor internalization may decrease the specificity of the pSTAT response in Type I IFN signaling. This effect is not an inherent property of receptor internalization, but rather is the result of the particular internalization rates observed in the Type I IFN system. These results also demonstrate that SOCS1 and receptor internalization have non-redundant roles as feedback mechanisms in Type I IFN signaling.

### No Evidence of Time-Course Signal Discrimination

**Figure 4:**
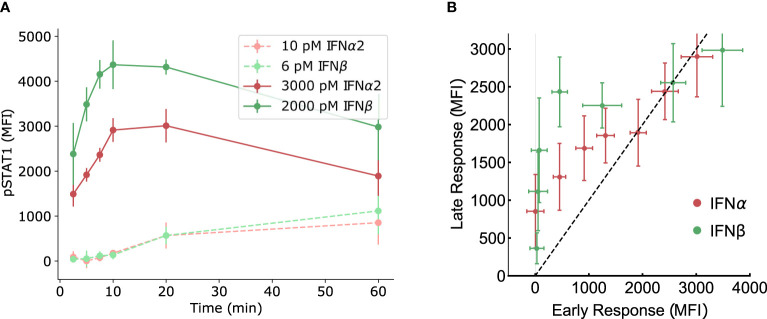
No evidence of time-course signal discrimination. **(A)** The same experimentally measured data from mouse B cells presented in **Figure 2D** is re-plotted here as time courses for IFN*α*2 and IFN*β* at comparably low (~10^1^ pM) and high (~10^3^ pM) doses. There is no apparent difference in the character (transient *versus* sustained) responses between IFN subtypes in either concentration regime. **(B)** The measured pSTAT response at early (5 minutes) and late (60 minutes) times, at equal concentrations, are plotted with the early-time response on the horizontal axis and late-time response on the vertical axis. A sustained response would produce points on the diagonal (black dashed line) while a transient response would produce points away from the diagonal. The points are not separable as transient *versus* sustained response based on the type of IFN, within the error bars.

Although we have not observed evidence of amplitude-based absolute discrimination in the pSTAT dose-response curves, absolute discrimination based on the time course of Type I IFN signaling must also be considered. The most common type of time course signal discrimination is based on distinguishing transient from sustained signaling dynamics (57–59), although more complicated inference is possible (79, 80). We investigate time course discrimination as a possible mechanism for distinguishing IFN*α*2 from IFN*β* by comparing early and late time levels of pSTAT. A sustained response exhibits similar pSTAT levels at both early and late times, while a transient response exhibits a different (usually diminished) pSTAT level at late times relative to early times. In **Figure 4A** we show that the character (transient or sustained) of the pSTAT response in our experiments appears to depend more on the concentration of IFN rather than the IFN identity, with a sustained response for low concentrations and a transient response for high concentrations. However, in either concentration regime, both IFNs seem to follow the same pattern, which suggests that the ligand discrimination does not occur *via* time course discrimination.

To investigate further, in **Figure 4B** we re-plot our pSTAT measurements in mouse B cells with the response at early times on the horizontal axis and the response at late times on the vertical axis (at different IFN concentrations). A sustained response would produce points on the diagonal (i.e., the line *pSTAT_early_* = *pSTAT_late_*) in such a plot, while a transient response would produce points away from the diagonal. Ligand discrimination would present itself in such a plot as a separability of points based on ligand identity. However, as **Figure 4B** shows, the data points corresponding to two different IFNs are not distinguishable (within the error bars) except perhaps for a narrow window of intermediate concentrations. Thus, we conclude that time course discrimination is unlikely to be important for response specificity in Type I IFN signaling although further studies might be required.

### Long Time Deactivation *via* USP18 Regulates Receptor Complex Stability to Achieve Absolute Discrimination

Our results thus far suggest that functional plasticity in Type I IFN signaling does not arise from absolute discrimination of the initial pSTAT response within the first 60 minutes. However, differential refractoriness in the pSTAT response mediated by the protein USP18 offers another potential mechanism to achieve absolute discrimination (52, 81). It has been established that the upregulation of USP18 in response to Type I IFN stimulation over the course of 24-48 hours leads to somewhere between a 15- and 60-fold increase in the in-membrane dissociation rate of IFNAR1 from the ternary complex, with the magnitude of this effect varying between cell types (10, 81). Inhibition by USP18 leads to significantly reduced response to further IFN stimulation, which is relevant for clinical uses of Type I IFN (10, 42, 81). Importantly, IFN*α*2 typically exhibits much greater refractoriness than IFN*β* at equivalent doses, although the degree of deactivation can be partially compensated by increasing the stimulation dose (42, 46). It is therefore possible that differential signaling mediated by USP18 over long timescales could lead to ligand specific differences in the *pSTAT_max_*, enabling absolute discrimination between the two ligands.

**Figure 5:**
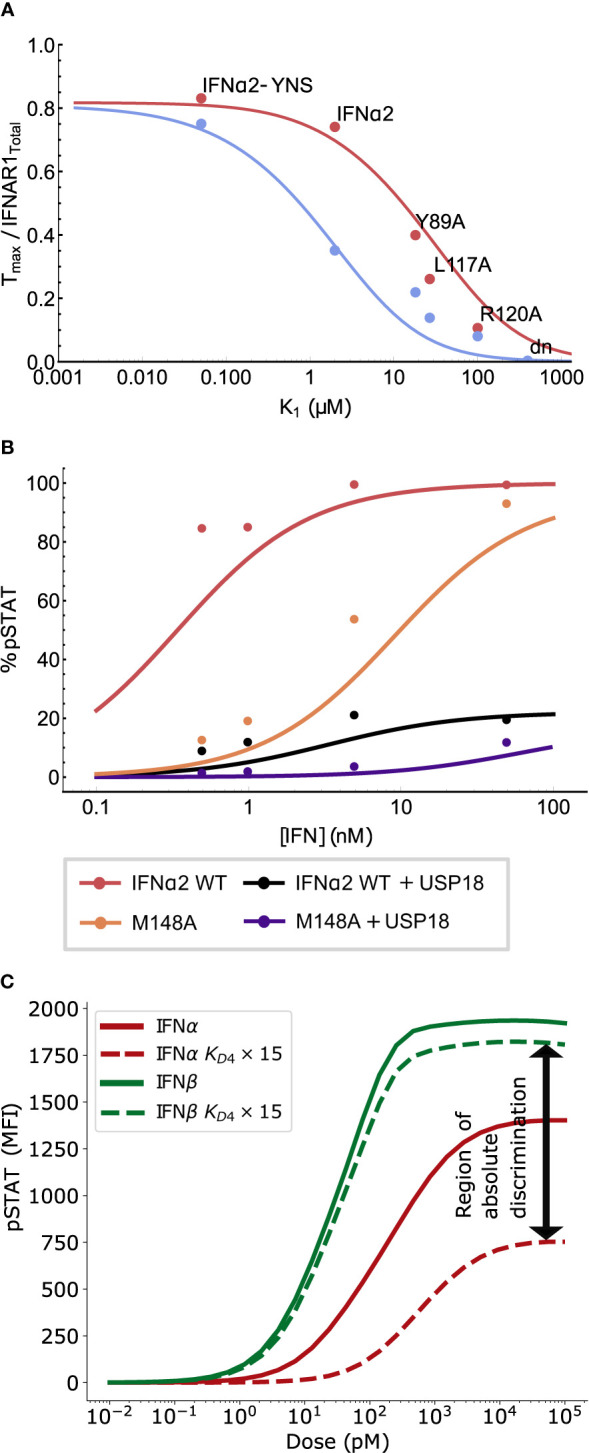
Long time deactivation *via* USP18 regulates receptor complex stability to achieve absolute discrimination. **(A)** Measurements reproduced from (10) of ternary complex formation in un-primed cells (red, also see **Figure 2A**) and cells primed with IFN to express USP18 (blue). Primed cells were observed to form fewer ternary complexes relative to unprimed cells. This reduction can be explained by a 15-fold increase in K4 in our analytic model (blue curve, produced from Equation 1); see text. **(B)** pSTAT1 Western blot band intensities for wild-type (WT) IFN*α*2 with and without USP18 expression, and IFN*α*2-M148A with USP18 expression are reproduced from (10). USP18 inhibition was modeled by a 60-fold increase in *K*_4_ in Eq. 4 (lines); see text. **(C)** The simulated effect of USP18 on the pSTAT response at 60 minutes for IFN*α*2 (red) *versus* IFN*β* (green). Solid lines show the un-primed response while dashed lines show the effect of a 15-fold increase in dissociation rate, consistent with the effect of USP18 (reported to be between 15- to 60-fold change depending on cell type). The differential refractoriness of IFN*α*2 as compared to IFN*β* greatly enhances the region of absolute discrimination (difference in *pSTAT_max_*).

Since the action of USP18 occurs on the intracellular domain of the receptor, its effects are represented in the model *via* a change in *K*_4_, the in-membrane dissociation constant of IFNAR1 binding to the binary complex IFN : IFNAR2 (see *Mathematical Methods*). **Figure 5A** shows that the experimentally observed decrease in the numbers of ternary complexes in cells primed with IFN compared with unprimed cells can be accounted for by a 15-fold increase in *K*_4_ (10). Crucially, the USP18-induced change in the in-membrane dissociation constant *K*_4_ not only shifts the dose response curve towards higher concentrations but also decreases the plateau saturation level *pSTAT_max_*, raising the possibility of amplitude-based absolute discrimination. The model also reproduces the effect of USP18 refractoriness on the mutant IFN*α*2-M148A, as shown in **Figure 5B**. The agreement between the model and the data validates the simple approach of modeling the presence of USP18 indirectly as a shift in *K*_4_, at least for high expression levels of USP18.

Interestingly, comparison of **Figure 2C** with **Figure 5B** shows that the effect of USP18 (which only alters *K*_4_) on the pSTAT dose-response curve is recapitulated by the IFN*α*2-R120A mutant that has lower binding strength to IFNAR1 (which alters both *K*_1_ and *K*_4_), as was first described in (10). The similarity of these two perturbations on the dose-response curve indicates that ternary complex formation is highly sensitive to the rate of in-membrane receptor subunit association.

We further examined how USP18 may affect the ligand discrimination specifically between IFN*α*2 and IFN*β*, as shown in **Figure 5C**, using our computational model. It demonstrates that the action of USP18 is predicted to reduce the amplitude of the IFN*α*2 dose-response curve much more than the corresponding amplitude of the IFN*β* dose-response, in agreement with previously reported experimental results (42) and our own experimental analysis (not shown). The reason for this differential refractoriness is that the high affinity of IFN*β* for IFNAR1 still allows formation of ternary complexes even in the presence of USP18, which is not the case for IFN*α*2 due to its lower affinity to IFNAR1. Most importantly, this differential effect of USP18 increases the separation in *pSTAT_max_* values between the two interferons and leads to the emergence of a region of absolute discrimination, which is expected to be apparent at long times (10-48 hours) when sufficient USP18 has accumulated in the cell. This fact will be important for the potential explanation of the differences in the anti-viral and anti-proliferative activities in Section *Proximal Receptor Signaling Maps to Biological Activity*.

### Proximal Receptor Signaling Maps to Biological Activity

Interestingly, the time scale for USP18 upregulation is similar to that for the emergence of anti-proliferative activity (~24 hours) (11, 48, 82). By contrast, anti-viral genes are typically upregulated very quickly (less than 8 hours) in response to IFN stimulation, regardless of IFN subtype (11, 83). Motivated by this observation, we now discuss how differential signaling at the level of pSTAT may be translated into differences in physiological activity, focusing on the hypothesis that the differential activity may be linked to the regulation of differential pSTAT response by USP18 mentioned in the last section and **Figure 5**.

A multitude of signaling reactions and gene expression downstream of receptor signaling are involved in anti-viral and anti-proliferative responses. Not all of these reactions are known, making detailed modeling of these processes unfeasible. Rather, in this paper we use phenomenological mappings that translate the pSTAT response into anti-viral and anti-proliferative activity, as measured by common experimental functional assays, to gain insights into the emergence of functional plasticity based on the specificity of the receptor and STAT phosphorylation levels (38–40). Despite substantial variability in the results of anti-viral and anti-proliferative assays between different studies, our conclusions remain qualitatively robust across cell types and experimental variation.

**Figure 6:**
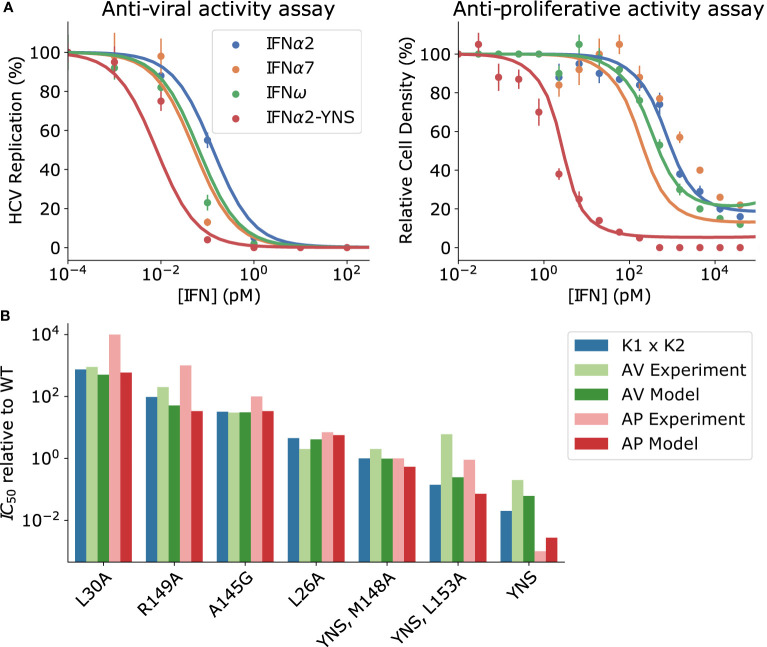
Proximal receptor signaling maps to biological activity. **(A)** Points: measurements of biological activity from (39). Lines: phenomenological mapping (Eq. 5-6). IFN*α*2-YNS is frequently used as an IFN*β* mimic because it has a similar affinity for IFNAR1 and IFNAR2 as wild-type IFN*β*. The antiviral activity was measured experimentally as the percentage of HCV-infected cells, while anti-proliferative activity was measured as the percentage of cells remaining alive after IFN stimulation for 48 hours. In both assays, maximal biological activity corresponds to minimal replication/cell density. **(B)** The anti-viral (AV) IC_50_ (green) and anti-proliferative (AP) IC_50_ (red) measured for a selection of IFN*α*2 mutants is compared with the predicted IC_50_’s from Equations 5 and 6 respectively. The product of IFNAR1 and IFNAR2 dissociation constants for each IFN subtype is shown in blue, and is a good phenomenological approximation to IC_50_; see *Mathematical Methods*.

Our aim is to capture the following notable features distinguishing anti-viral and anti-proliferative activity. First, the anti-viral *IC*_50_ (the IFN concentration required for half-maximal anti-viral activity) is substantially lower than the *pSTAT EC*_50_ [**Figure 6A** (left)]; this is not the case for anti-proliferative activity [**Figure 6A** (right)] (38, 39). Second, complete inhibition of viral replication is achievable by all IFN subtypes that have been investigated while the maximum inhibition of cell proliferation differs between IFN subtypes by as much as ~20% (38–40).

The observed discrepancy between the *IC*_50_ of the anti-viral response and the *pSTAT EC*_50_ indicates that the components of the JAK-STAT pathway downstream of pSTAT that mediate anti-viral gene transcription require very little pSTAT to become activated. We found that despite its unusual properties, the dependence of the anti-viral response on the pSTAT levels can be reproduced by a simple Michaelis-Menten type dependence:


(5) $$\text{Anti‐ viral activity}\propto \frac{pSTAT(IFN)}{K_{M}+pSTAT(IFN)}$$

where *pSTAT(IFN)* is the intracellular concentration of pSTAT as a function of the extracellular IFN concentration, and *K_M_* is the effective Michaelis-Menten constant for the intra-cellular processes leading to anti-viral activity. As shown in **Figure 6A**, anti-viral activity described by Eq. 5 saturates at much lower IFN concentrations than the corresponding pSTAT dose-response curve (c.f. **Figure 2D**) for sufficiently small *K_M_* (i.e., *K_M_* ~pM); we used *K_M_* = 0.8*pM* in **Figure 6A**.

The anti-viral *IC*_50_ can be found by substituting Eq. 4 for the pSTAT response into Eq. 5 and solving for the IFN concentration which gives half-maximal activity. In the limit of *K_M_*/[*S_T_*] ≪1, the *IC*_50_ is proportional to (*pSTAT EC*_50_) × *K_M_*/[*S_T_*], recalling that [*S_T_*] is the concentration of intracellular STAT (our model used [*S_T_*] = 0.7 nM). It follows from this proportionality that a small *K_M_* guarantees that the anti-viral *IC*_50_ is lower than the *pSTAT EC*_50_. This also explains why the anti-viral *IC*_50_ in this regime is essentially the same for all IFNs because differences in *pSTAT EC*_50_ between IFNs are also shrunk by the factor *K_M_*/[*S_T_*].

We now turn to anti-proliferative activity. A phenomenological mapping for anti-proliferative activity must preserve differences in maximal anti-proliferative response between IFN subtypes, as apparent in the high concentration regime of **Figure 6A** (right), as well as the difference in the anti-proliferative *IC*_50_ between IFN*β* and the other IFN subtypes [also **Figure 6A** (right)]. These differences between IFNs are only observed in anti-proliferative assays, not anti-viral assays. Since the anti-proliferative activity emerges on longer time scales we include the effect of USP18 on the pSTAT response, denoted as *pSTAT_primed_* (*IFN*) (29, 31, 52). Unlike the anti-viral response that could be modeled by a single Michaelis-Menten type function, the anti-proliferative response required a more complex function of the following form to explain the data in **Figure 6B**:


(6) $$\text{Anti‐ proliferative activity}\propto \frac{1}{2}\frac{{\left(pSTAT_{primed}(IFN)\right)}^{\gamma_{1}}}{{\left(K_{M1}\right)}^{\gamma_{1}}+{\left(pSTAT_{primed}(IFN)\right)}^{\gamma_{1}}}+\frac{1}{2}\frac{{\left(pSTAT_{primed}(IFN)\right)}^{\gamma_{2}}}{{\left(K_{M2}\right)}^{\gamma_{2}}+{\left(pSTAT_{primed}(IFN)\right)}^{\gamma_{2}}},$$

where *γ*_1_ and *γ*_2_ are phenomenological Hill coefficients and *K_M_*_1_, *K_M_*_2_ are the effective anti-viral *EC*_50_’s for each contributing term. Fitting Eq. 6 to the data, using our model of pSTAT induction presented in Section *Validation of the Model and the Effects of Ligand Affinity on Response Specificity* and measured IFN binding affinities (39), results in the best fit parameter values *K_M_*_1_ = 72 *pM*, *K_M_*_2_ = 158 *pM*, *γ*_1_ = 0.9, *γ*_2_ = 3.7. For sufficiently large values of *K_M_*_1_ and *K_M_*_2_ – on the order of 100 pM – differential refractoriness expressed in the different saturation levels between the strong binding IFN*α*2-YNS and the weaker binding variants results in the differential saturation of anti-proliferative activity at high concentrations in **Figure 6A** (right). Thus, absolute discrimination on the pSTAT level shown in **Figure 5C** translates into functional plasticity on the level of anti-viral and anti-proliferative responses. This result is robust to variation in the parameters of Eq. 5-6, not simply the result of a choosing specific parameter values.

To demonstrate the robustness of our phenomenological mappings, in **Figure 6B** we compare the predicted IC_50_ values of both the anti-viral and anti-proliferative responses to previously reported measurements for several IFN*α*2 mutants spanning four orders of magnitude in *K_eff_* (39). We find that our phenomenological models semi-quantitatively recapitulate both anti-viral and anti-proliferative IC_50_’s for most IFNs, although agreement was somewhat worse for the quadruple mutant IFN*α*2-YNS-L153A as well as for the weakest binding IFNs IFN*α*2-L30A and IFN*α*2-R149A. The product *K*_1_ × *K*_2_ using affinities measured for each IFN is provided in **Figure 6B** for context, and provides a good estimate of each *IC*_50_ other than the high affinity mutant IFN*α*2-YNS (39). This quantity has been used as an approximation for *K_eff_* in previous work, and we show in **Figure S5** that this approximation is reasonable for a realistic range of *K*_1_ and *K*_2_. The advantage of our phenomenological mappings is that they can predict the biological activity at any concentration, and the maximal biological activities, for each IFN subtype; the quantity *K*_1_ × *K*_2_ alone cannot provide such estimates.

The order of magnitude difference between *K_Mi_* for anti-proliferative activity (~100 pM as obtained from the best fit values for Eq. 6) and *K_M_* for anti-viral activity (less than 1 pM), as well as the Hill coefficients greater than 1 in Eq. 6, suggest that different molecular processes are responsible for the activation of these two types of cellular response. We return to this point in the Discussion.

## Discussion

Different Type I IFN subtypes induce distinct cellular responses despite the fact that all Type I IFNs bind to the same receptor. This functional plasticity arises from a combination of biophysical and dynamic factors and is at least partially decoupled from ligand concentration, indicating the presence of absolute discrimination in this signaling system (29, 38–40). In this paper we have presented a model of Type I IFN signaling validated with previously published and our own experimental data in a variety of cell types. This model accurately captures signaling dynamics both at the level of receptor assembly and at the level of pSTAT induction. Our validated model demonstrates that many molecular details of this system can be aggregated into single step reactions with effective rate constants, increasing interpretability without losing predictive power. Predictions of the model are in agreement with a more detailed model, further indicating that our coarse-grained minimal model that subsumes many molecular details into effective parameters is sufficient to capture the dynamics of the pSTAT response and the emergence of functional plasticity.

The main objective in this paper – beyond producing a realistic and generalizable model of Type I IFN signaling - was to investigate the mechanisms of ligand discrimination (among several primary candidates) that can explain functional plasticity in Type I IFN signaling (Sections *No Evidence of Time-Course Signal Discrimination; Long Time Deactivation via USP18 Regulates Receptor Complex Stability to Achieve Absolute Discrimination*). The first candidate mechanism considered was amplitude-based absolute discrimination, which encodes ligand identity in the saturation magnitude of the downstream response and enables functional plasticity by thresholding activity based on the level of response (53). The second candidate mechanism, time-course discrimination, achieves functional plasticity by conditioning the cellular response on the temporal pattern of signaling activity in response to different ligands (59). A third mechanism, combinatorial encoding, was not studied in this work (6, 61). The three mechanisms are not mutually exclusive. For example, amplitude discrimination which emerges only at late times in the signaling dynamics is conceptually related to time course discrimination by distinguishing transient from sustained responses. However, each mechanism focuses on a different aspects of signal processing: specificity across concentrations, or through time.

We showed that a difference in the ligand binding strength can theoretically generate amplitude-based absolute discrimination by inducing a difference in *pSTAT_max_*, and indeed this has been observed for the mutant IFN*α*2-R120A and in other signaling pathways (Section *Validation of the Model and the Effects of Ligand Affinity on Response Specificity*). However, no region of absolute discrimination was observed between IFN*α*2 and IFN*β* in experiments. In practice, specificity in Type I IFN signaling appears to emerge primarily due to the action of the long-time negative feedback protein USP18 which is expressed in response to IFN stimulation. USP18 effectively increases the in-membrane dissociation of ternary complex, and the much weaker receptor binding strength of IFN*α*2 as compared to IFN*β* leads to a greater inhibitory effect on the IFN*α*2 response, thus generating a difference in the *pSTAT_max_* between these IFNs (Section *Long Time Deactivation via USP18 Regulates Receptor Complex Stability to Achieve Absolute Discrimination*).

We also used our model of Type I IFN signaling as a basis for a phenomenological understanding of both anti-viral and anti-proliferative activities across the entire spectrum of IFN binding affinities. In Section *Proximal Receptor Signaling Maps to Biological Activity* we showed that a Michaelis-Menten function with a *K_M_* of only a few picomolar maps the pSTAT response to anti-viral activity for several IFNs, which suggests that maximal anti-viral gene transcription requires only a few copies of pSTAT. A small *K_M_* is also consistent with observations that all IFN subtypes can completely block viral replication, because in this case even the weakest binding IFNs can induce enough pSTAT to reach saturation in Eq. 5.

In contrast to the *K_M_* of anti-viral activity, the larger *K_M_*_1_ and *K_M_*_2_ used in our anti-proliferative phenomenological mapping requires a greater accumulation of pSTAT to saturate the functional response. Differences in the maximal anti-proliferative activity between IFNs then arise due to differences in their *pSTAT_max_* amplified by USP18 inhibition. This suggests that USP18 may provide a mechanism for cells to differentially regulate their anti-proliferative activity, without altering their potential for an anti-viral response. Furthermore, a Hill coefficient greater than one suggests molecular cooperativity or other complex regulation of anti-proliferative activity downstream of pSTAT (84, 85). Our phenomenological models point to different intracellular signal processing for anti-viral functions as compared to anti-proliferative functions, consistent with previous suggestions (32, 86).

Combinatorial encoding is an alternative mechanism for absolute discrimination, not investigated here, in which the set of signaling components involved encode the ligand identity and enable a specific response (87, 88). This mechanism is particularly effective for responding differently to different combinations of extracellular ligands (6, 89, 90), but can also be used to achieve absolute discrimination in crosstalk signaling systems (61). Reports of functional roles for pSTAT1 homodimers and complexes involving pSTAT3 or pSTAT5 may indicate combinatorial encoding, and future investigation may reveal additional complexities in signal processing for Type I IFNs (39, 82, 86).

We showed in Sections *Receptor Expression Levels Can Regulate Signaling Specificity; The Role of Negative Feedbacks in Signal Specificity* that differences in *pSTAT_max_* can be tuned by a variety of feedbacks. There is evidence that receptor internalization and recycling rates differ between cell types, so it is possible that receptor internalization is an important factor differentiating the response to IFN stimulation in different cell types (36). Additionally, we noted that total receptor expression could be regulated to ensure that some cell types only exhibit responses associated with high affinity IFNs. Other feedbacks we did not consider here include signaling from endocytosed receptor complexes and hypersensitization of the pSTAT response with low level IFN pre-stimulation (11, 36, 81). Feedback from ISG upregulation at longer timescales such as a reduction in IFNAR1 surface levels or activity of Tyk2 can be studied in our framework in a similar fashion to feedback by SOCS1, USP18, or receptor internalization studied in this work (Sections *Receptor Expression Levels Can Regulate Signaling Specificity; The Role of Negative Feedbacks in Signal Specificity; Long Time Deactivation via USP18 Regulates Receptor Complex Stability to Achieve Absolute Discrimination*), and will be investigated in future work. While we did not find it necessary to model these effects to explain the emergence of functional plasticity, such mechanisms may play (possibly redundant) roles in some cell types. Whether or not signal processing of Type I IFNs differs between cell types is an interesting but open question.

Finally, our models for Type I IFN signaling can be used as a framework for engineering functional properties of IFNs for improved clinical use. For example, refractoriness to repeated IFN treatment can be a serious challenge for clinical applications (42, 81). Our results suggest that a non-refractory version of IFN*α*2 could be engineered with a similar pSTAT1 *EC*_50_ to the wild type IFN by increasing the IFNAR1 affinity and simultaneously decreasing IFNAR2 affinity. More generally, the approach presented here provides a broadly applicable framework for studying response specificity in systems involving crosstalk downstream of the receptor or combinatorial sensing of cytokine mixtures.

## Data Availability Statement

The original contributions presented in the study are included in the article/**Supplementary Material**. Further inquiries can be directed to the corresponding author.

## Ethics Statement

The animal study was reviewed and approved by the institutional animal care and use committee (IACUC) of the National Cancer Institute (Bethesda MD, USA).

## Author Contributions

AZ, GA-B, JF, and SMac were responsible for conceptualization, funding acquisition, supervision, resources, and project administration. All authors contributed to methodology and investigation. DK was responsible for formal analysis, visualization, software, methodology, validation. DK and AZ wrote the paper. All authors contributed to the article and approved the submitted version.

## Funding

AZ acknowledges the support of Natural Sciences and Engineering Research Council (NSERC) of Canada through the Discovery Grant (RGPIN 402591). DK acknowledges the support of the PGS-D Graduate Fellowship from NSERC. GAB acknowledges support from the Intramural Research Program of the National Institutes of Health (USA). 

## Conflict of Interest

The authors declare that the research was conducted in the absence of any commercial or financial relationships that could be construed as a potential conflict of interest.

## Publisher’s Note

All claims expressed in this article are solely those of the authors and do not necessarily represent those of their affiliated organizations, or those of the publisher, the editors and the reviewers. Any product that may be evaluated in this article, or claim that may be made by its manufacturer, is not guaranteed or endorsed by the publisher.
