# Supplementary material for: Determinants of Ligand Specificity and Functional Plasticity in Type I Interferon Signaling
Source: Front Immunol. 2021 Oct 7;12:748423. doi: 10.3389/fimmu.2021.748423 (PMC8529159; doi:10.3389/fimmu.2021.748423)
Supplement: Supplementary file 1 [file DataSheet_1.pdf]

Supplementary Materials

Code for the computational IFN model can be found at: https://doi.org/10.5281/zenodo.5507232

Antibody specificity	Label	Clone	Target specie	Host species	Supplier
CX3CR1	BV421	SA011F11	mouse	mouse	Biolegend
I-A/I-E	BV480	M5/114	mouse	rat	BD
NK1.1	BV650	PK136	mouse	rat	BD
F4/80	BV711	BM8	mouse	rat	Biolegend
Ly6G	BV785	1A8	mouse	rat	BD
pSTAT1	-	58D6	mouse	rabbit	Cell Signaling
FAb anti-Rabbit	FITC	polyclonal	rabbit	donkey	Jackson Immunochemicals
CD19	PE	1D3	mouse	rat	Tonbo
CD3e	PE-Cy5	145-2C11	mouse	rat	Biolegend
CD4	APC	RM4-5	mouse	rat	Tonbo
CD8a	APC-Cy7	53-6.7	mouse	rat	Tonbo

Table S1. Antibodies used to stain primary mouse splenocytes.

Table S2 and Table S3 list the complete set of ordinary differential equations and associated rate constants used to model Type I IFN signaling.

**Equation**	**Species changing**
$\mathrm{IFN}'_{\alpha}[t] = \mathrm{B1}_{\alpha}{:}\mathrm{SOCS}[t] * \mathrm{kd1}^{\alpha} + \mathrm{B1}_{\alpha}[t] * \mathrm{kd1}^{\alpha} + \mathrm{B2}_{\alpha}[t] * \mathrm{kd2}^{\alpha} - \mathrm{IFN}_{\alpha}[t] * \mathrm{R1}{:}\mathrm{SOCS}[t] * \mathrm{ka1}^{\alpha} - \mathrm{IFN}_{\alpha}[t] * \mathrm{R1}[t] * \mathrm{ka1}^{\alpha} - \mathrm{IFN}_{\alpha}[t] * \mathrm{R2}[t] * \mathrm{ka2}^{\alpha}$	Free IFNα2
$\mathrm{IFN}'_{\beta}[t] = \mathrm{B1}_{\beta}{:}\mathrm{SOCS}[t] * \mathrm{kd1}^{\beta} + \mathrm{B1}_{\beta}[t] * \mathrm{kd1}^{\beta} + \mathrm{B2}_{\beta}[t] * \mathrm{kd2}^{\beta} - \mathrm{IFN}_{\beta}[t] * \mathrm{R1}{:}\mathrm{SOCS}[t] * \mathrm{ka1}^{\beta} - \mathrm{IFN}_{\beta}[t] * \mathrm{R1}[t] * \mathrm{ka1}^{\beta} - \mathrm{IFN}_{\beta}[t] * \mathrm{R2}[t] * \mathrm{ka2}^{\beta}$	Free IFNβ
$\mathrm{R1}'[t] = \mathrm{kSOCSoff} * \mathrm{R1}{:}\mathrm{SOCS} + \mathrm{kd1}^{\beta} * \mathrm{B1}_{\beta} + \mathrm{kd4}^{\beta} * \mathrm{T}_{\beta} + \mathrm{kd1}^{\alpha} * \mathrm{B1}_{\alpha} + \mathrm{kd4}^{\alpha} * \mathrm{T}_{\alpha} + \mathrm{kreca1} * \mathrm{R1}_{\mathrm{int}} + \mathrm{krecr1} * \mathrm{R1}_{\mathrm{int}} - \mathrm{kIntBasalr1} * \mathrm{R1} - \mathrm{kSOCSon} * \mathrm{R1} * \mathrm{SOCS} - \mathrm{ka1}^{\beta} * \mathrm{IFN}_{\beta} * \mathrm{R1} - \mathrm{ka4}^{\beta} * \mathrm{B2}_{\beta} * \mathrm{R1} - \mathrm{ka1}^{\alpha} * \mathrm{IFN}_{\alpha} * \mathrm{R1} - \mathrm{ka4}^{\alpha} * \mathrm{R1} * \mathrm{B2}_{\alpha}$	IFNAR1
$\mathrm{R2}'[t] = \mathrm{kd2}^{\beta} * \mathrm{B2}_{\beta} + \mathrm{kd3}^{\beta} * \mathrm{T}_{\beta} + \mathrm{kd3}^{\beta} * \mathrm{T}_{\beta}{:}\mathrm{SOCS} + \mathrm{kd2}^{\alpha} * \mathrm{B2}_{\alpha} + \mathrm{kd3}^{\alpha} * \mathrm{T}_{\alpha} + \mathrm{kd3}^{\alpha} * \mathrm{T}_{\alpha}{:}\mathrm{SOCS} + \mathrm{kreca2} * \mathrm{R2}_{\mathrm{int}} + \mathrm{krecr2} * \mathrm{R2}_{\mathrm{int}} - \mathrm{kIntBasalr2} * \mathrm{R2} - \mathrm{ka2}^{\beta} * \mathrm{IFN}_{\beta} * \mathrm{R2} - \mathrm{ka3}^{\beta} * \mathrm{B1}_{\beta}{:}\mathrm{SOCS} * \mathrm{R2} - \mathrm{ka3}^{\beta} * \mathrm{R2} * \mathrm{B1}_{\beta} - \mathrm{ka2}^{\alpha} * \mathrm{IFN}_{\alpha} * \mathrm{R2} - \mathrm{ka3}^{\alpha} * \mathrm{B1}_{\alpha}{:}\mathrm{SOCS} * \mathrm{R2} - \mathrm{ka3}^{\alpha} * \mathrm{R2} * \mathrm{B1}_{\alpha}$	IFNAR2
$\mathrm{STAT}'[t] = \mathrm{pSTAT}[t] * \mathrm{kpu} - \mathrm{T}_{\alpha}[t] * \mathrm{STAT}[t] * \mathrm{kpa} - \mathrm{Tbeta}[t] * \mathrm{STAT}[t] * \mathrm{kpa}$	STAT1
$\mathrm{pSTAT}'[t] = \mathrm{T}_{\alpha}[t] * \mathrm{STAT}[t] * \mathrm{kpa} + \mathrm{T}_{\beta}[t] * \mathrm{STAT}[t] * \mathrm{kpa} - \mathrm{pSTAT}[t] * \mathrm{kpu}$	Phospho-STAT1
$\mathrm{SOCS}'[t] = \mathrm{R1}{:}\mathrm{SOCS}[t] * \mathrm{kSOCSoff} + \mathrm{B1}_{\alpha}{:}\mathrm{SOCS}[t] * \mathrm{kSOCSoff} + \mathrm{B1}_{\beta}{:}\mathrm{SOCS}[t] * \mathrm{kSOCSoff} + \mathrm{T}_{\alpha}{:}\mathrm{SOCS}[t] * \mathrm{kSOCSoff} + \mathrm{T}_{\beta}{:}\mathrm{SOCS}[t] * \mathrm{kSOCSoff} + \mathrm{pSTAT}[t] * \mathrm{kSOCS} - \mathrm{SOCS}[t] * \mathrm{SOCSdeg} - \mathrm{B2}_{\beta}[t] * \mathrm{SOCS}[t] * \mathrm{kSOCSon} - \mathrm{T}_{\alpha}[t] * \mathrm{SOCS}[t] * \mathrm{kSOCSon} - \mathrm{T}_{\beta}[t] * \mathrm{SOCS}[t] * \mathrm{kSOCSon} - \mathrm{R1}[t] * \mathrm{SOCS}[t] * \mathrm{kSOCSon} - \mathrm{SOCS}[t] * \mathrm{B1}_{\alpha}[t] * \mathrm{kSOCSon}$	SOCS
$\mathrm{B1}'_{\alpha}[t] = \mathrm{kSOCSoff} * \mathrm{B1}_{\alpha}{:}\mathrm{SOCS} + \mathrm{ka1}^{\alpha} * \mathrm{IFN}_{\alpha} * \mathrm{R1} + \mathrm{kd3}^{\alpha} * \mathrm{T}_{\alpha} - \mathrm{kd1}^{\alpha} * \mathrm{B1}_{\alpha} - \mathrm{kSOCSon} * \mathrm{SOCS} * \mathrm{B1}_{\alpha} - \mathrm{ka3}^{\alpha} * \mathrm{R2} * \mathrm{B1}_{\alpha}$	IFNAR1:IFNα2
$\mathrm{B2}'_{\alpha}[t] = \mathrm{IFN}_{\alpha}[t] * \mathrm{R2}[t] * \mathrm{ka2}^{\alpha} + \mathrm{T}_{\alpha}[t] * \mathrm{kd4}^{\alpha} - \mathrm{B2}_{\alpha}[t] * \mathrm{kd2}^{\alpha} - \mathrm{R1}[t] * \mathrm{B2}_{\alpha}[t] * \mathrm{ka4}^{\alpha}$	IFNAR2:IFNα2

$B1'_{\beta}[t] = kSOCSoff * B1_{\beta}: SOCS + ka1^{\beta} * IFN_{\beta} * R1 + kd3^{\beta} * T_{\beta} - kd1^{\beta} * B1_{\beta} - kSOCSon * SOCS * B1_{\beta} - ka3^{\beta} * R2 * B1_{\beta}$	IFNAR1:IFNβ
$B2'_{\beta}[t] = IFN_{\beta}[t] * R2[t] * ka2^{\beta} + T_{\beta}[t] * kd4^{\beta} - B2_{\beta}[t] * kd2^{\beta} - B2_{\beta}[t] * R1[t] * ka4^{\beta}$	IFNAR2:IFNβ
$R1: SOCS'[t] = B1_{\alpha}: SOCS[t] * kd1^{\alpha} + B1_{\beta}: SOCS[t] * kd1^{\beta} + T_{\beta}: SOCS[t] * kd4^{\beta} + T_{\alpha}: SOCS[t] * kd4^{\alpha} + R1[t] * SOCS[t] * kSOCSon - R1: SOCS[t] * kSOCSoff - IFN_{\alpha}[t] * R1: SOCS[t] * ka1^{\alpha} - IFN_{\beta}[t] * R1: SOCS[t] * ka1^{\beta} - R1: SOCS[t] * B2_{\alpha}[t] * ka4^{\alpha} - R1: SOCS[t] * B2_{\beta}[t] * ka4^{\beta}$	IFNAR1:SOCS
$R1'_{int}[t] = R1[t] * kIntBasalr1 + T_{\alpha,int}[t] * kdega + T_{\beta,int}[t] * kdegb - R1_{int}[t] * kreca1 - R1_{int}[t] * krecr1$	Internalized IFNAR1
$R2'_{int}[t] = T_{\alpha,int}[t] * kdega + T_{\beta,int}[t] * kdegb + R2[t] * kIntBasalr2 - R2_{int}[t] * kreca2 - R2_{int}[t] * krecr2$	Internalized IFNAR2
$B1_{\alpha}: SOCS'[t] = IFN_{\alpha}[t] * R1: SOCS[t] * ka1^{\alpha} + T_{\alpha}: SOCS[t] * kd3^{\alpha} + SOCS[t] * B1_{\alpha}[t] * kSOCSon - B1_{\alpha}: SOCS[t] * kSOCSoff - B1_{\alpha}: SOCS[t] * kd1^{\alpha} - B1_{\alpha}: SOCS[t] * R2[t] * ka3^{\alpha}$	IFNAR1:IFNα2:SOCS
$B1_{\beta}: SOCS'[t] = IFN_{\beta}[t] * R1: SOCS[t] * ka1^{\beta} + B1_{\beta}[t] * SOCS[t] * kSOCSon + T_{\beta}: SOCS[t] * kd3^{\beta} - B1_{\beta}: SOCS[t] * kSOCSoff - B1_{\beta}: SOCS[t] * kd1^{\beta} - B1_{\beta}: SOCS[t] * R2[t] * ka3^{\beta}$	IFNAR1:IFNβ:SOCS
$T'_{\alpha}[t] = T_{\alpha}: SOCS[t] * kSOCSoff + R1[t] * B2_{\alpha}[t] * ka4^{\alpha} + R2[t] * B1_{\alpha}[t] * ka3^{\alpha} - T_{\alpha}[t] * kd3^{\alpha} - T_{\alpha}[t] * kd4^{\alpha} - T_{\alpha}[t] * kinta - T_{\alpha}[t] * SOCS[t] * kSOCSon$	IFNAR1:IFNα2:IFNAR2
$T_{\alpha}: SOCS'[t] = R1: SOCS[t] * B2_{\alpha}[t] * ka4^{\alpha} + B1_{\alpha}: SOCS[t] * R2[t] * ka3^{\alpha} + T_{\alpha}[t] * SOCS[t] * kSOCSon - T_{\alpha}: SOCS[t] * kSOCSoff - T_{\alpha}: SOCS[t] * kd3^{\alpha} - T_{\alpha}: SOCS[t] * kd4^{\alpha}$	IFNAR1:IFNα2:IFNAR2:SOCS
$T'_{\beta}[t] = B2_{\beta}[t] * R1[t] * ka4^{\beta} + T_{\beta}: SOCS[t] * kSOCSoff + R2[t] * B1_{\beta}[t] * ka3^{\beta} - T_{\beta}[t] * kd3^{\beta} - T_{\beta}[t] * kd4^{\beta} - T_{\beta}[t] * kintb - T_{\beta}[t] * SOCS[t] * kSOCSon$	IFNAR1:IFNβ:IFNAR2
$T_{\beta}: SOCS'[t] = B1_{\beta}: SOCS[t] * R2[t] * ka3^{\beta} + T_{\beta}[t] * SOCS[t] * kSOCSon - T_{\beta}: SOCS[t] * kSOCSoff - T_{\beta}: SOCS[t] * kd4^{\beta} - T_{\beta}: SOCS[t] * kd3^{\beta}$	IFNAR1:IFNβ:IFNAR2:SOCS
$T'_{\alpha,int}[t] = T_{\alpha}[t] * kinta - T_{\alpha,int}[t] * kdega$	Internalized IFNAR1:IFNα2:IFNAR2
$T'_{\beta,int}[t] = T_{\beta}[t] * kintb - T_{\beta,int}[t] * kdegb$	Internalized IFNAR1:IFNβ:IFNAR2

Table S2. A complete description of ODEs used to model the pSTAT response to IFN.

Parameter	Value	Source or Notes
$ka1^{\alpha}$	$3.8 \times 10^5\ M^{-1}s^{-1}$	Mouse IFNα2 affinity measured in Ref. (1), used in Figure 2D-E, Figure 3F-H, Figure 5C
$ka1^{\alpha}$	$2 \times 10^5\ M^{-1}s^{-1}$	Human IFNα2 affinity measured in Ref. (2), used in Figure 2A-C, Figure 3A-E, Figure 5A-B, and Figure 6
$kd1^{\alpha}$	$1\ s^{-1}$	(2)
$ka2^{\alpha}$	$4.6 \times 10^8\ M^{-1}s^{-1}$	Mouse IFNα2 affinity measured in Ref. (1)
$ka2^{\alpha}$	$4 \times 10^6 M^{-1}s^{-1}$	Human IFNα2 affinity measured in Ref. (2)
$kd2^{\alpha}$	$0.015\ s^{-1}$	(2)
$ka3^{\alpha}$	$ka4^{\alpha}$	
$kd3^{\alpha}$	$0.007\ s^{-1}$	From detailed balance
$ka4^{\alpha}$	$4\pi \times 0.094$ molecules^{-1} μm^{-2} s^{-1}	(3)
$kd4^{\alpha}$	0.29 molecules μm^{-2} $\times\ ka4^{\alpha}$	(3)
$ka1^{\beta}$	$7.9 \times 10^7\ M^{-1}s^{-1}$	Mouse IFNβ affinity measured in Ref. (1)
$ka1^{\beta}$	$4 \times 10^5 M^{-1}s^{-1}$	Human IFNβ affinity measured in Ref. (2)

kd1 $^{\beta}$	$0.03\ s^{-1}$	(2)
ka2 $^{\beta}$	$6 \times 10^{5}\ M^{-1}s^{-1}$	Mouse IFNβ affinity measured in Ref. (1)
ka2 $^{\beta}$	$1 \times 10^{7}\ M^{-1}s^{-1}$	Human IFNβ affinity measured in Ref. (2)
kd2 $^{\beta}$	$0.002\ s^{-1}$	(2)
ka3 $^{\beta}$	ka3 $^{\alpha}$	
kd3 $^{\beta}$	$2.4*10^{-5}s^{-1}$	From detailed balance
ka4 $^{\beta}$	ka4 $^{\alpha}$	
kd4 $^{\beta}$	$0.002\ s^{-1}$	$=$ ka4 $^{\beta} \times \mathrm{K}^{\alpha}{}_{4}\ \mathrm{K}^{\beta}{}_{1}\ /\mathrm{K}^{\alpha}{}_{1}$
μ_{R1} & μ_{R2}	2 000 molecules	Mean IFNAR1/2 expression, fit by MCMC; Similar to (4)
STAT	10^{4} molecules	Initial value
kp+	$1 \times 10^{-6}s^{-1}$	STAT1 phosphorylation rate, fit by MCMC
kp-	$0.95 \times 10^{-3}s^{-1}$	STAT1 dephosphorylation rate
σ_{R1} & σ_{R2}	0.2 molecules	Std. dev. of receptor expression, fit by MCMC
SOCS	1 molecule	Initial value
kSOCS	$4 \times 10^{-3}s^{-1}$	SOCS1 synthesis rate
SOCSdeg	$25 \times 10^{-4}s^{-1}$	SOCS1 degradation rate
kSOCSon	$6 \times 10^{-7}s^{-1}$	Rate of SOCS1 binding to IFNAR1, fit by MCMC
kSOCSoff	$5.5 \times 10^{-4}s^{-1}$	Rate of SOCS1 unbinding from IFNAR1
kIntBasalr1	$1 \times 10^{-4}s^{-1}$	Basal rate of IFNAR1 internalization, estimated from (5)
kIntBasalr2	$2 \times 10^{-5}s^{-1}$	Basal rate of IFNAR2 internalization, estimated from (5)
krecr1	$1 \times 10^{-4}s^{-1}$	Rate of IFNAR1 recycling back to cell surface, estimated from (5)
krecr2	$1 \times 10^{-4}s^{-1}$	Rate of IFNAR2 recycling back to cell surface, estimated from (5)
kdega	$8 \times 10^{-4}s^{-1}$	Rate at which internalized IFNAR1:IFNα:IFNAR2 is disassembled, estimated from (5)
kdegb	$8 \times 10^{-4}s^{-1}$	Rate at which internalized IFNAR1:IFNβ:IFNAR2 is disassembled, estimated from (5)
kinta	$5.2 \times 10^{-4}s^{-1}$	Rate at which IFNAR1:IFNα:IFNAR2 is internalized, fit by MCMC
kintb	$5.2 \times 10^{-4}s^{-1}$	Rate at which IFNAR1:IFNβ:IFNAR2 is internalized, fit by MCMC
kreca1	$1 \times 10^{-3}s^{-1}$	Rate of additional IFNAR1 recycling back to cell surface, fit by MCMC
kreca2	$0.1\ s^{-1}$	Rate of additional IFNAR1 recycling back to cell surface, fit by MCMC
kbind	$10^{-6}\ s^{-1}$ molecules	Rate of STAT1 binding to ternary complex
kunbind	$4\ s^{-1}$	Rate of STAT1 unbinding from ternary complex
kin	$1.25 \times 10^{-4}\ s^{-1}$	Rate of pSTAT1 localization to nucleus
kout	$0.6\ s^{-1}$	Rate of pSTAT1 localization to cytoplasm
kmRNA	$10^{-3}\ s^{-1}$	Rate of SOCS1 mRNA synthesis from ISRE
kexp	$10^{-3}\ s^{-1}$	Rate of mRNA localization to cytoplasm
kprot	kSOCS	Rate of SOCS1 synthesis from mRNA

Table S3. Rate constant prior values for ODE model of pSTAT response to IFN. Parameter values taken from the literature are within experimental errors. All parameters were fit with log-normal priors centered at the values in the table and using scale parameter 0.2, except for μ_{R1}, μ_{R2} which were fit using scale parameter 1.0, and σ_{R1} and σ_{R2} which used scale parameter 0.1. Parameters used exclusively in the ODE model (not shared with the equilibrium model) are shaded light gray. Additional parameters used in the detailed model of Figure S3 are shaded dark gray.

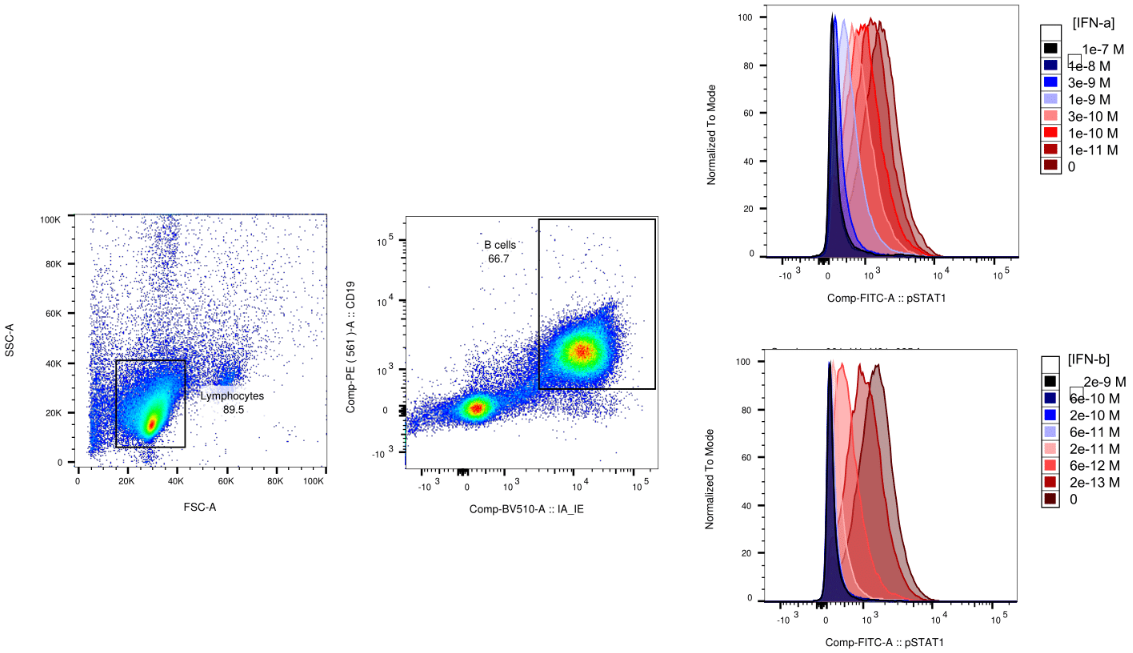

Figure S1. Gating strategy for B cells from mouse splenocytes.

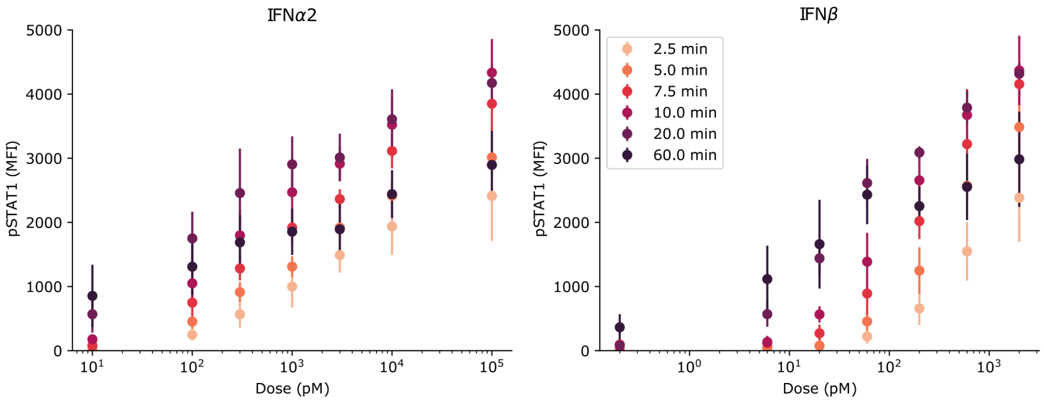

Figure S2. The pSTAT1 mean fluorescence intensity measured for all concentrations and time points tested. This is the data which the ODE model was fit to.

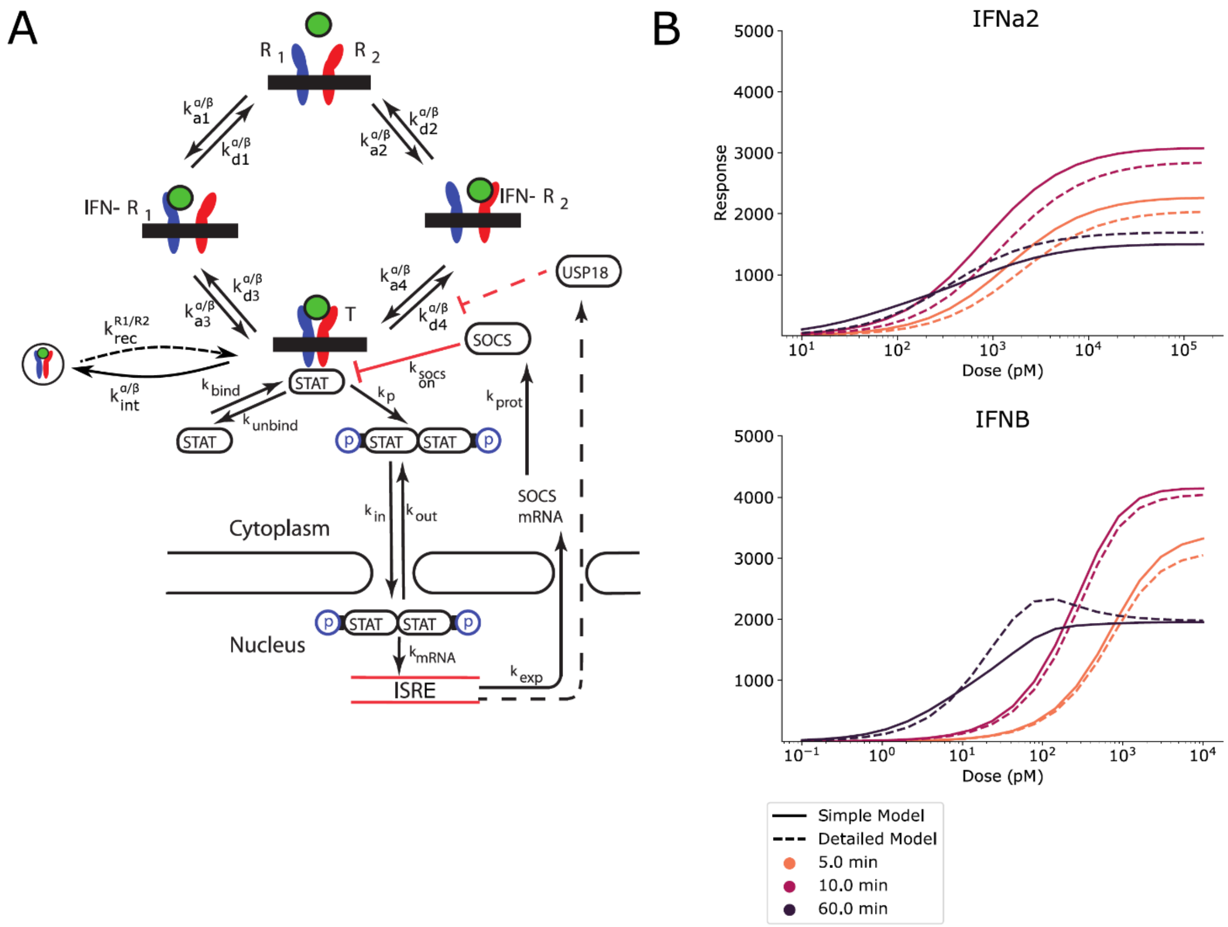

Figure S3. A detailed model of Type I IFN signaling captures similar signaling dynamics. **A)** The detailed model explicitly represents STAT binding and unbinding to the receptor as separate steps in addition to STAT phosphorylation. Transcription factor translocation to the nucleus and mRNA transcription and translation to produce SOCS are also modeled as separate steps. **B)** The dose response curves of the detailed (dashed) and simple (solid) models are very similar. Explicitly modeling each of the additional steps of the detailed model does not change any of the results presented in the main paper.

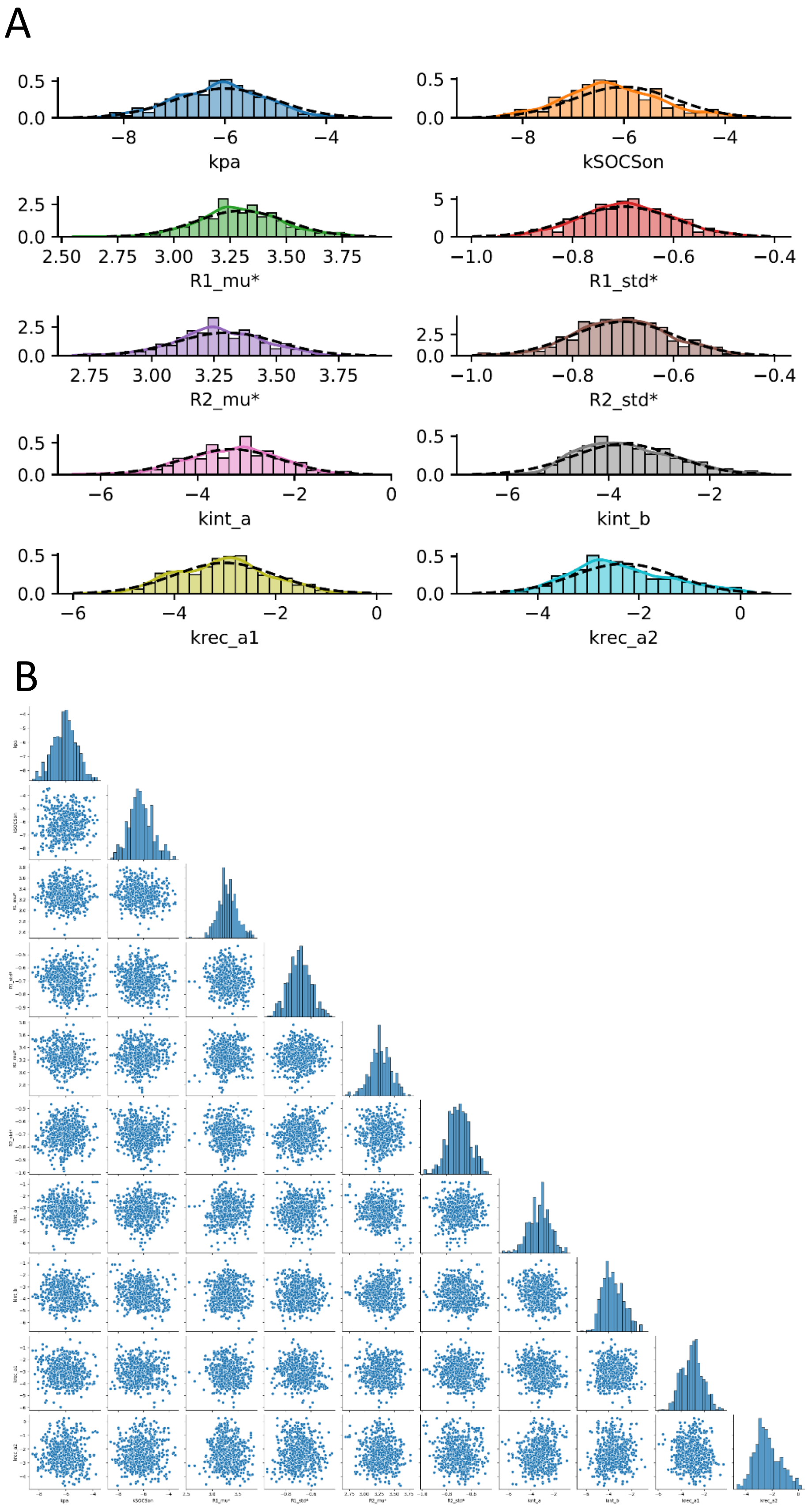

Figure S4.

Results of fitting the computational model to data using MCMC.

A) The marginal parameter distributions found by MCMC. Many parameters are not significantly different from their prior distributions (black dashed lines). The parameters with * in the name are associated with modeling the distribution of IFNAR expression levels to be sampled.

B) A corner plot showing pairwise marginal distributions found by MCMC. No significant pairwise correlations are observed, indicating that fitted parameters are not tightly constrained by each other.

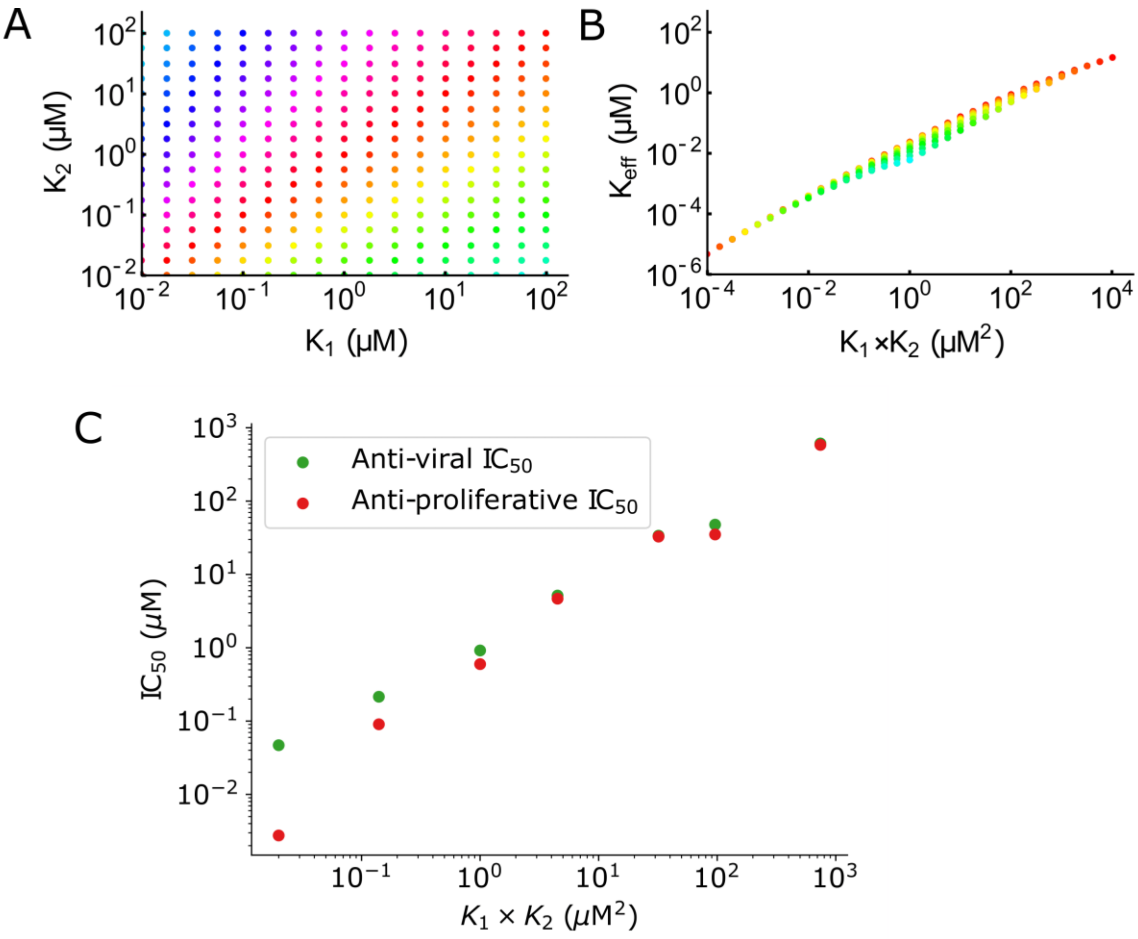

Figure S5. Approximating K_{eff} using $K_1 \times K_2$. **A)** The points from the parameter space (K_1, K_2) are coloured as shown and the same points are used in (B). **B)** The product $K_1 \times K_2$ is plotted against K_{eff} from Eq. 3, using the values and colours of K_1 and K_2 shown in (A), $K_4 = K_4^{\alpha 2\,WT} \times \frac{K_1}{K_1^{\alpha 2\,WT}}$, $R_T = 2000$, and $\Delta = 0$. K_{eff} is nearly linearly related to $K_1 \times K_2$ for a wide range of affinities, meaning it is expected to exhibit the same correlation with pSTAT response and biological activity that K_{eff} exhibits. There is a systematic bias in using $K_1 \times K_2$ since the true K_{eff} is about 10^{-2} smaller than $K_1 \times K_2$. **C)** The model IC_{50} values for each IFN (see also Figure 6B) versus the product of dissociation constants as reported in (6). The product $K_1 \times K_2$ does correlate with each IC_{50}, as expected from (B).

Supplementary Information References

1. S. A. Stifter, *et al.*, Defining the distinct, intrinsic properties of the novel type i interferon, IFNϵ. *J. Biol. Chem.* **293**, 3168–3179 (2018).

2. D. A. Jaitin, *et al.*, Inquiring into the Differential Action of Interferons (IFNs): an IFN-α2 Mutant with Enhanced Affinity to IFNAR1 Is Functionally Similar to IFN-β. *Mol. Cell. Biol.* **26**, 1888–1897 (2006).

3. S. Wilmes, *et al.*, Receptor dimerization dynamics as a regulatory valve for plasticity of type I interferon signaling. *J. Cell Biol.* **209**, 579–593 (2015).

4. F. Kok, *et al.*, Disentangling molecular mechanisms regulating sensitization of interferon alpha signal transduction. *Mol. Syst. Biol.* **16**, 1–32 (2020).

5. Z. Marijanovic, J. Ragimbeau, J. van der Heyden, G. Uzé, S. Pellegrini, Comparable potency of IFNα2 and IFNβ on immediate JAK/STAT activation but differential down-regulation of IFNAR2. *Biochem. J.* **407**, 141–151 (2007).

6. C. Thomas, *et al.*, Structural linkage between ligand discrimination and receptor activation by type I interferons. *Cell* **146**, 621–632 (2011).
